# Analectic

**Published:** 1836

**Authors:** 


### MISCELLANEOUS INTELLIGENCE.

---

### ANALECTIC, ANALYTICAL, AND ORIGINAL.

---

### I.—ANALECTIC.

---

*Ad Sylvas Nuncius.*

---

1. *On the Ligature of Arteries in case of Aneurism.* By Dr. Placido Portal.—One of the most important operations of clinical surgery in the present state of the science, is that of tying the arteries in case of aneurism; and in my estimation we should account every surgeon extremely fortunate who should perform it with success. So grave and so numerous are the failures, and so uncertain the final success, that the celebrated Crampton remarked, "whatever may be the method employed in tying the artery, the operation for aneurism is full of uncertainty and danger; it is seldom that the operator is free from anxiety with respect to the event; the patient always incurs great danger, and however skilful may be the operator, the number of fatal cases exceeds that of the successful." This is easily explained if we reflect upon the insurmountable obstacles which the most profound and experienced European surgeons have encountered; although they have constantly devoted their thoughts and studies to the discovery of the most secure method of obliterating the arteries in case of aneurism, and of preventing in season the ulcerative process and the secondary hemorrhage, yet by far the greater part of the patients have perished, whatever may have been the method employed for tying the artery, or the material of which the ligature was composed; and this, too, in spite of all their endeavors, and the researches of the celebrated Astley Cooper, Kline, Blizard, Freer, Pearson, Hodgson, Abernethy, etc., etc., not to speak of many professors of the Neapolitan school. "We do not know (repeated the celebrated clinical professor of Pisa) how to avoid with safety the hemorrhage consequent upon the operation for aneurism, which arises perhaps not unfrequently from an abnormal state of our solids, and perhaps in some degree of our fluids."

For the happy success of the operation, proper precautions in tying the artery are of equal importance with a simple and speedy mode of operating, and to ensure the former it is necessary to bear in mind the following conditions; there should be a moderate division of the parts, and as little drawing upon the artery as possible; no rupture of its coats, and a proper degree of irritation and adhesive inflammation; since nature proceeds first to form adhesion between the parietes of the tied artery, and then to cicatrize the retracted parts. This truth, so well known to all practitioners, has induced the more enterprising surgeons to search for a mechanical method, which should not excite a greater degree of inflammation than should be circumscribed within the limits of the adhesion. In the accomplishment of this object, some surgeons, wishing to avoid one of the greatest inconveniences, the secondary hemorrhage, make use of a number of ligatures placed a short distance from each other, with the design of increasing the points of contact, and retarding by degrees the force of the blood from the point where the artery had been secured. Others, following the instructions of Celsus, believe the safer plan to be to tie the artery with two ligatures, and to cut between. Some, under the belief that it is necessary to divide the internal and middle coats of the artery, to procure with more facility the adhesive inflammation, advise the ligature with a simple strand of silk. Others are loud in praising the use of a single ligature, to *avoid* rupturing these coats, and for this purpose interpose a piece of linen stiffened with wax between the ligature and artery. Some propose to maintain the mutual contact of the parietes of the artery by metallic compressors constructed for the purpose. And others, finally, apply fine ligatures of silk to different points of the artery, and afterwards dividing them close to the knot, suffer them to remain in the wound.

Each of the favorers of the above methods boasts his own experience and observation in support of it, since in the operations performed by them the artery was observed to be perfectly impervious, sufficient lymph being thrown out in 46 hours to cause the adhesive inflammation of the internal or middle coats, as I myself observed in some experiments performed by me upon dogs and sheep in 1824; but does this imply that the secondary hemorrhage is certainly prevented? I answer, that simple pressure is sufficient to produce adhesion of the arterial parietes; but that a strong pressure may in some cases cause a rupture of the membranes, ulceration and secondary hemorrhage.

At the close of February, 1827, I was called to a young man named Philip Ramaglia, of Palermo, aged 24, who had imprudently wounded the left radial artery under the styloid apophysis of the radius. He had been under the care of a surgeon who had stopped the hemorrhage by compression, but upon the second day after, the blood flowed in such quantity as to alarm the patient and his friends. I removed the compresses and applied a ligature upon the artery half an inch above where it had been wounded. The seventh day after, so profuse a discharge of blood took place, that the event would have been fatal if I had not applied immediate compression to the mouth of the bleeding vessel. On removing the dressing, I perceived that the artery had been ulcerated by the ligature, and thought it necessary to tie it once more half an inch above the point where it had been ruptured, leaving a ligature upon the artery in reserve. The twelfth day after the second operation, hemorrhage again recurred, and more violently than at first, and I was obliged to tighten the other ligature which I had left on the artery, which I did gently; the hemorrage ceased soon after, and I observed no trickling of blood. The ninth day after the ligature came away, and on the thirtieth the patient was perfectly cured.

Another case, more important than the last, took place in the August of 1830, in a man named Antonia Randazzo, 50 years of age, of a bilious idiosyncrasy. He received a gunshot wound in his left arm, which partly severed the brachial artery. I had tied it three times in different parts, but unluckily the ulcerative process which supervened gave rise to dreadful hemorrhages, perhaps because the artery itself was in an abnormal state. I persevered, however, and by tying it at the point where this artery leaves the axillary, and moderating the pressure of the ligature, I succeeded in uniting it; the ligature came off on the thirteenth day, and the patient was perfectly cured.

Instructed by the preceding observations, and by similar ones which I subsequently made in the military hospitals, and the civil hospital of Palermo, I have not since used tight ligatures. In the March of 1834, I, in the same way, tied the right femoral artery of his Reverence D. Camillo Starvaggi, 70 years of age, Chaplain of the Parish of Brancaccio. The operation was crowned with complete success; the artery united on the fourth day, the ligature came away on the eighth, and the patient was perfectly well on the fourteenth.

I am thus induced to conclude that the too tight application of the ligature contributes in no way to the success of the operation, since the adhesive process will always go on independently of it, if the artery be in a healthy condition.

With the above difficulties in view, great credit is due to Prof. Mansella for having taken up the external iliac in a case of aneurism, in the inferior third of the right thigh, in which the femoral artery was in an abnormal state. The account of the operation, as related by Dr. Carmelo, his son, with the morbid phenomena which accompanied it, is as follows.

"The subject of this operation was a soldier 36 years of age, of a sanguineous temperament and a strong constitution. On the 12th of March, 1834, he entered the hospital with an aneurismal tumor in the inferior third of the right thigh. On the 29th a consultation was held upon the expediency of an operation, which Dr. Mansella performed upon the same day, taking up the artery at its upper third, and securing it with a permanent ligature. The patient remained perfectly calm till the 13th of April, the 17th day after the operation, when an alarming hemorrhage ensued from the ruptures of the tied artery, which however was quickly arrested by pressure applied by the tourniquet of Petit. In the night the hemorrhage returned, and on the 14th, while changing the dressings, was so profuse as to threaten the life of the patient.

"Mansella immediately called another consultation of all the surgeons of the establishment, who concurred with him in the necessity of tying the external iliac as the only hope left of avoiding another hemorrhage, which would certainly destroy the patient. The operation was performed by Mansella on the same day. At first the patient was in great fear lest hemorrhage should again recur; but seeing the aneurismal tumor diminish sensibly in size, his fortitude returned. On the 24th of April, the ligature came away, the wound gradually united, the suppuration diminished, and on the 14th of May he was discharged from the hospital perfectly cured."*—*Filliatre Sebezio, a Journal of Medical Sciences, March,* 1835.—*Boston Medical and Surgical Journal,* No. 9.

2. Anatomy.—*On the Arteries of the Corneal Coujunctiva,* By Dr. Romer, of Vienna.—This paper commences with a full and impartial account of the various opinions which have been entertained by different anatomists regarding the covering of the cornea. A very simple experiment, viz. throwing the eye of an animal into boiling water, shows that the cornea is covered externally by a membrane, which, although it be continued into the conjunctiva, differs remarkably from the latter tunic in physical properties, and is as strikingly different from the proper substance of the cornea. The membrane in question appears to be albuminous; for in this experiment it immediately coagulates and becomes opaque, while the palpebral and sclerotic portions of the conjunctiva suffer no such change. Dr. Romer regards the conjunctiva as a continuation of the common integuments, changed into mucus membrane on the inside of the eyelids, and again, on the anterior part of the sclerotica and on the cornea, transformed into serous membrane. He speaks of the Meibomian and caruncular follicles as the sebaceous glands of the conjunctiva; but, to our view, the Meibomian follicles open through the skin, not through the conjunctiva. It is well known that that portion of the conjunctiva which covers the internal surface of the tarsi or cartilages is villous or papillous, while the remainder of the membrane is comparatively smooth. It is the villi of the former which are so much enlarged and indurated in granular conjunctiva. They are considered by Eble† not as secreting organs, but merely as nervous papillæ, like those of the skin or of the tongue; but the general notion is that they are mucus glands, and this appears to be the opinion also of Dr. Romer.

In the normal state, says Dr. Romer, the conjunctiva lining the inner surface of the eyelids is moderately red, while that portion which covers the eyeball is of a pearl color, and only towards the angles of the eye presents occasionally some insulated red serpentine streaks, which are blood-vessels. After a tolerably successful injection, the conjunctiva of the eyelids presents a high red color and a peculiar velvety appearance, bearing a striking resemblance to the

---

*Prof. Velpeau, in his Operative Medicine, vol. 1, page 87, says that the external iliac artery has been tied 41 times successfully for aneurism in different parts of Europe.

†Ueber den Bau und die Krankheiten der Bindehaut des Auges. Wien, 1828.

Schneiderian membrane covering the septum narium, while from the ocular conjunctiva the pearly hue has vanished, and in its stead we find a rose-red color, reaching in general only as far as the edge of the cornea. In a completely successful injection, the vessels are prolonged to the centre of the cornea, where they evidently penetrate and are lost in its substance.

The vessels which are distributed to that part of the conjunctiva which covers the anterior portion of the sclerotica and the cornea, are derived, as well as those which belong to the palpebral conjunctiva, from the lacrymal, the superior and inferior palpebral, and the oculo-muscular arteries. The small branches of these arteries form in that part of the conjunctiva which covers the anterior portion of the sclerotica, a superficial and a deep-seated network. The superficial network is derived from numerous branches arising from the superior and inferior palpebra arteries, and from the lacrymal. Dividing, as they proceed, into smaller branches, they run in a serpentine course towards the edge of the cornea, where they anastomose together in arches, and connect themselves with the deep-seated net-work. The deep-seated net-work is formed by much smaller vessels, partly derived from the oculo-muscular arteries, and partly from the ciliary, before these penetrate through the sclerotica. The ramifications of the two net-works form at the edge of the cornea a vascular wreath, which lies just over the circular venous sinus of the iris. From every part of this wreath arise numerous minute ramifications, running towards the centre of the cornea, each dividing, as they run, into two or three still smaller vessels, which plainly dip into the substance of the cornea.

[We regard Dr. Romer's paper as a valuable contribution to the anatomy of the eye.]—*Zeitschrift fur die Ophthalmologie*, B. v. H. I. 1835.—*British and Foreign Medical Review, July*, 1836.

3. *On the Power by which the Head of the Thigh-bone is retained in its Socket.* By Dr. Edward Weber.—[At the meeting of German naturalists at Bonn, 25th September, 1835, a paper on this subject was read by Dr. Weber; and, on the following day, the experiments referred to in it were repeated before the Anatomico-Zoological Section of the Association. We shall give a translation of the greater part of this interesting paper, omitting merely the account of the experiments performed at the meeting, which were identical with those described in Dr. Weber's communication.]

It has been generally believed that it is through the power of the muscles or ligaments that the head of the thigh-bone is retained in its socket; and this, probably, because they are the first to strike our senses. A closer investigation, however, has proved that it is neither the muscles nor ligaments that effect this, but the pressure of the atmosphere. The head of the thigh-bone, enclosed in its air-tight cavity, remains, in its place, on the same principle that the piston remains fixed in a cylinder, the upper extremity of which is closed with the finger; being forced by the air against the upper walls of its cavity, just as the mercury in the barometer is forced upwards by the same agent. The following experiments prove this fact:

1st. The trunk of the body was laid on a table in such a position that the leg and thigh hung down unobstructed. If the bone were supported in its place by the muscles and ligaments, it must clearly fall when the muscles and ligaments are divided. I divided the muscles and ligaments, and the limb did *not* fall: on the contrary, the bones constituting the joint remained in close contact, as before.

2d. If we admit that the limb is maintained in its position by atmospheric pressure, it is clear that it should fall down when air is admitted into the joint. I bored a hole through the wall of the bony cavity of the joint, and on the admission of the air the limb fell; and the displacement followed on boring the hole, even when the muscles and ligaments were not divided.

3d. If we assume that the atmospheric pressure is *alone* sufficient to maintain the limb in its place, the bone which had fallen down ought to be retained there when replaced and the air excluded. I replaced the bone which had been severed from the body, and excluded the air from the joint by closing the artificial opening with the finger; the limb remained supported in its place; and immediately fell down once more on the finger being removed.

I will only here refer to one instance of the practical application of this view of the mechanism of the thigh-joint,—the case known by the name of *spontaneous luxation of the thigh*. It is well known that, in this case, the bone is displaced without any obvious external cause, the limb being at first considerably lengthened, with marked lameness.

Since it has been shown that the head of the bone is not retained in its place by the power of the ligaments, it is not therefore necessary, for the explanation of the case, to admit that the ligaments must, previously to its occurrence, be lengthened, or (to speak generally) *altered*, in any way. Our experiments have shown that the head of the bone fell immediately from its socket into the inferior portion of the capsular ligament (in die hohle der kapsel herabsank,) upon the admission of air into the cavity of the joint. But, to produce this result, it is not necessary that air should be introduced from without; the same effect will follow the secretion of a fluid or the growth of a solid body in the cavity of the joint itself. In proportion as such a substance, whether fluid or solid, accumulates, the head of the bone will necessary fall down through its own weight, without the application of any force, and without the least resistance being opposed by the ligaments of the joint.

[The experiments at the Association were witnessed, among others, by Leuckart, Mayer, Schultz, Munke, and the two Webers; all of whom were convinced of the retention of the bone in its place by the pressure of the atmosphere.]—*Muller's Archiv. fur Anatomie, etc. Jahrgang*, 1836. Heft. 1.—*Ib.*

4. Medicine, Pathological, Practical, and Therapeutical.—*On Thymic Asthma.* By Dr. Hirsch, of Kœnigsberg.—[Dr. Kopp read a paper on this subject at the annual meeting of scientific men at Heidelberg, which has since been published in a separate work by the author, (*Denkwurdigkeiten in der artzlichen Praxis.* Bd. i. *Frankfurt*, 1830.) In the present memoir Dr. Hirsch, has collected some additional cases, and has drawn up a digested account of the whole disease from collation of the various memoirs published on the subject in Germany, since Dr. Kopp first called particular attention to it. We shall give, in the first place an abridgement of Dr. Kopp's original cases, and then an abstract of Dr. Hirsch's general observations.]

Case 1. A delicate woman, with weak lungs, who had suffered from atony of the uterus after a confinement with her sixth child, was brought to bed, for the seventh time, of a puny male infant, which, notwithstanding it was well fed, continued extremely emaciated. From its birth to its death, it frequently held its breath. This difficulty in breathing was at first hardly remarked, but it increased extremely, and came on particularly on waking, swallowing, or crying. It died in the seventh month, in a fit of suffocation with convulsions. An examination was not made, nor did the case make much impression on Dr. Kopp, until he had seen two similar cases in the children of the same mother.

Case 2. A brother of the first infant, born at the full term, having a delicate constitution, but enjoying good health for the first four months, began from that period to be affected similarly, by a suspension of respiration at intervals, attended each time with a slight plaintive cry, coming on suddenly, and evidently producing pain. After gaining its breath, it testified by its cries the pain and anxiety it had suffered. During the paroxysm, the pulse was irregular and intermitting, eyes fixed, hands and feet cold, face puffed and bluish, and tongue pushed out between the teeth. The attacks increased in frequency and violence, but with this exception the health was not impaired. After an attack of catarrh when the child was ten months old, they increased greatly, and the child died in one of the paroxysms in a state of asphyxia; the face puffed and blue, and the tongue thrust between the teeth.

*Examination, twenty-two hours after death.* Tongue rather long, and thickened at the root; trachea healthy; thyroid gland tumefied; sanguineous extravasation covering the trachea at the junction of the thyroid and thymus glands. Thymus so large and thick, that an assistant took it for a lobe of the lung; extending from the thyroid gland to the diaphragm; two inches broad, weighing more than an ounce, and pressing against the trachea, where the effusion had taken place; on cutting it, much milky fluid, which had penetrated its whole substance, escaped; it was nowhere indurated. Lungs brownish red, gorged with blood, as in asphyxia. Heart flabby and atrophied. Foramen ovale open.

Case 3. Twenty months after the death of the last child, the mother was confined with another boy, of a similar constitution, but rather stronger. Fifteen weeks after its birth, the same symptoms as in the other two cases commenced, so that the mother recognized the disease; there was also spasmodic contractions of the hands and feet. The treatment consisted in leeches to the epigastrium, calomel, lavements of valerian and musk, frictions with white precipitate, which caused an eruption followed by some amelioration. Under these means the nervous symptoms diminished, and after taking six baths of infusion of chamomile and valerian, (which were discontiuued, as they exhausted,) the paroxysms diminished in frequency, so that many days were passed without any. On examining the chest, the pulsation of the heart could not be discovered. Sometime afterwards, without any known cause, there were slight spasms of the hands and feet, with abdominal pain, contraction and slight swelling of the features, and protrusion of the tongue. Flowers of zinc, musk, ipecacuanha, and calomel, were of temporary benefit; and sometimes the paroxysm was shortened by laying the child on its face, and gently patting its back. The child however emaciated, its digestion was affected, and it vomited and had whitish and greenish stools; the paroxysms returned with violence, and in one of them it died asphyxiated, in the twenty-second week of its existence.

*Examination, twenty-seven hours after death.* Thymus very large, occupying the whole anterior part of the thorax, and adhering strongly to its superior part; it touched the thyroid gland, and covered the heart; it weighed an ounce; it was infiltrated with a milky fluid. Tongue large, thick, and protruded between the teeth. Glottis free; trachea healthy; heart flabby; foramen ovale closed; right lung gorged with blood; pulmonary tissue otherwise healthy, and floating in water. Cerebral substance slightly softened; abdominal viscera sound.

The two following cases were communicated to Dr. Kopp by Dr. Rullman, and they were both children of the same mother.

Case. The disease began during the third week, and the symptoms were exactly similar to those detailed by Dr. Kopp. In the twenty-first month the child, whilst playing, stooped to pick up something he had dropped; he was immediately attacked with vertigo, and fell backwards into his father arms; his face turned red and blue, his limbs became rigid, his skin pale, the stools and urine escaped involuntarily, and the child died suffocated. On dissection, it was found that the thymus extended from the upper border of the sternum to the diaphragm, covering not only the trachea and pericardium, but the whole anterior surface of the lungs. Its texture was rather firmer than usual, granulated, and infiltrated with a milky fluid. Lungs small, compressed, and gorged with blood, but otherwise healthy.

The other case was more fortunate. The same symptoms came on about the third month, and were aggravated at the sixth month, owing to dentition. As Dr. Rullman was aware of the cause, he adopted more suitable means; he prescribed Plummer's pill (in powder) with hemlock, applied a blister to the sternum, recommended low diet fresh air, and the avoidance of any sources of disquietude. Gastric or catarrhal symptoms were carefully combatted. During the first eight weeks there was no improvement, and the symptoms were aggravated whenever a tooth was cut; then purgative doses of calomel were useful. From this time the symptoms improved, and at the end of the second year the child was well. He is now nine years old, and quite healthy.

[The memoir of Dr. Hirsch contains five cases which are so similar to the foregoing ones, in every important particular, that we shall not give the details. The first was in a female infant, and no examination took place; the other four were boys. Two of these died, and the thymus was found enlarged, the other two lived. The treatment consisted in applying leeches and counter-irritants to the sternums and giving calomel and rhubarb internally. In one case, small doses of Aqua lauri cerasi and musk were also prescribed. We shall now pass on to the general history of the disease, treatment, etc., as collected by Dr. Hirsch from his own cases and those of others.]

It is characterised by attacks of spasm of the chest and severe fits of suffocation. The breathing suddenly stops, or rather there is an extremely slight, piping, imperfect inspiration, forced, as it were, through the contracted glottis. The respiratory sound has some resemblance to the crowing inspiration of hooping-cough, but is much smaller and more acute; it is still more like the choking attempts at inspiration made during the hysteric spasm. In some cases, but rarely, there may be five or six piping or whistling inspirations, and then a few deeper and stronger, alternating with expirations so slight as scarcely to be perceived. In extreme attacks, the respiration stops *entirely;* the small inspiratory pipe then takes place, either in the beginning of the paroxysm or in its termination, being quite suppressed during the strength of the attack; and this symptom is pathognomic of the affection. The other symptoms are necessary consequences of the suppressed respiration; the body is extended forcibly backwards or the limbs drawn close up together; the face, which is fixed and expressive of great distress, is either purple or quite pale; the nostrils are expanded, the eyes fixed, the hands cold, the thumbs contracted, with involuntary dejection, etc. After a period of half a minute or a minute, occasionally even two or three minutes, the paroxysm ceases; the infant then cries for a short time, and immediately becomes cheerful and easy. When the constitution is very feeble, however, or the attack has been very severe, the child remains some time languid, pale, and sleepy. In the intervals of the paroxysms the child is quite well, without the slightest affection of the respiration. Kopp says that the tongue still continues to be thrust out more than is natural, but this is certainly not always the case. The attacks come on chiefly when the child awakes, at other times after crying or fretting, or from any cause that excites the lungs to increased action. At first the attacks are not frequent, coming only once in eight or more days; but they grow progressively more frequent, till they come on ten or twenty times in one day. In this stage, death often takes place in the paroxysm, although the little patient had been playing about a minute before, apparently in the most perfect health. More frequently, however, another stage succeeds before death, characterised by general convulsions of an epileptic kind; the convulsions sometimes alternating with; sometimes accompanying, the asthmatic paroxysms. In the intervals, the lumbrical muscles of the palm and the adductors of the thumb are sometimes permanently contracted, so as to maintain a rounded form of the hand. In these cases the child is carried off in a paroxysm, with symptoms both of suffocation and apoplexy, or quite suddenly, without any affection of the chest or prolonged agony of any kind.

On examination, besides the general appearances always following death from suffocation,—such as blueness of the skin, congestion of blood in the lungs and brain, etc.,—the thymus gland is always found considerably enlarged. The degree of enlargement varies extremely, as does the natural size of the gland. At times it is enlarged chiefly in length and breadth, more commonly, however, in thickness; in the last case the lungs are frequently thrust back by it into the posterior part of the chest; on other occasions, the gland is found closely connected with, and even surrounding, the large arteries and venous trunks of the thorax or neck. The texture of the gland is either quite normal or (what is more common) somewhat firmer and more fleshy and redder, but without the least trace of induration, suppuration, tubercalization, or other form of degeneration. In cases where the gland has been weighed, it has been found to vary from six or seven up to fourteen drachms. However, it would seem to have been still larger in some cases where it was not weighed.

The duration of the asthma of Kopp is very various, depending generally on the severity of the attacks: it commonly lasts some months. Children of a scrofulous habit are particularly subject to it, and boys especially, if indeed not exclusively. All diseases of the respiratory organs predispose to it,—such as catarrh, bronchitis, croup, hooping-cough, measles. Teething also predisposes to it.

Thymic asthma is far from being a rare disease, and has been noticed by many writers, who have given it other names. The principal of these is Marsh, who describes it under the name of *spasm of the glottis*, Kellie, Porter, Pretty, Richter, and perhaps North. Even the enlargement of the thymus gland as a cause of asthma in children has been recognized by several authors previously to Kopp, and particularly by Hood. (*Edinb. Journ. of Med. Sc.*, vol. iii.)

*Nature of the Disease.* It is an affection peculiar to early infancy, and consists of a periodical tonic spasm of the lungs, larynx, and glottis, perhaps also of the heart, eventually extending to the whole nervous system, under the form of epileptic convulsions. In it the thymus gland is more or less enlarged, but otherwise not diseased, and presses on the heart, bronchi, and large vascular trunks, impeding their functions. When, on examination after death, such an enlargement and abnormal compression exist, one need not seek further for proofs of the nature of the disease. Yet there have been many arguments adduced against the accuracy of this judgment, of which the following are the principal:

1. Great enlargement of the thymus has been found, without the presence of asthma.

2. The affection termed the asthma of Kopp has been observed when the thymus was not enlarged.

3. An organic disease cannot occasion periodical attacks, leaving intervals of perfect health.

4. The enlarged thymus ought to excite symptoms of cardica disease, while the suffocation in the disease of Kopp is evidently the result of constriction of the trachea and glottis.

5. The course, frequent cure, and occasional relief from antispasmodic remedies, are incompatible with the existence of an organic disease.

6. The enlarged thymus may be a consequence of the asthma, and not the cause.

*Prognosis.* The disease is always dangerous, but not desperate, particularly if the child is strong, and not disposed to catarrhal affections; also when the case is recent, the spasms not frequent nor severe, and no general convulsions has taken place.

*Treatment.* The following are the principal indications:

1. In the attack nothing more can be done than to place the child in a proper position, to rub its spine, perhaps to dash cold water on it, etc.

2. After the attack, to give antispasmodics, preparatory to the employment of means, directed to the chief pathological state.

3. To diminish and prevent the recurrence of all undue congestion and nervous excitement in the heart and lungs, by low diet, large and frequent local bleedings (every four or eight days,) blisters and issues on the chest, constant powerful purgatives, etc.

4. To lessen the size of the thymus by anti-scrofulous, resolving medicines,—such as mercury, iodine, etc.

[We have been induced to present to our readers the foregoing account of the affection there denominated *Thymic Asthma*, with the view of calling the attention of the profession to a more accurate investigation of its nature and all its causes, and not because we subscribe to the general accuracy of the statements and opinions therein contained. It is hardly necessary to inform the English reader that the affection noticed in the present paper was long known in this country previously to the publication of Dr. Kopp. This is indeed admitted by Dr. Hirsch; and all pretence to originality in the views of Dr. Kopp respecting is cause its removed by the reference made by him to the paper of Mr. Hood, in the Edinburgh Journal of Medical Science, published in January, 1827. In this paper the existence of the enlarged thymus in such cases is proved by numerous dissections, and recognised by the author as a cause of the disease. The title of *Koppian Asthma*, bestowed on this disease by Dr. Hirsch, is evidently improper; and that of *Thymic Asthma*, given to it by Dr. Kopp himself, is no less so; since it can be demonstrated to have often existed without any morbid enlargement of the thymus gland. At the same time we do not doubt that such an enlargement is occasionally present, and operates as an exciting cause of the affection. The whole subject assumes at this moment a more striking interest from the publication of Dr. Ley's work on the same curious affection, and denominated by him *Laryngismus stridulous*, of which notice is taken in another part of this Number. We may refer our junior readers for a neat epitome of the history of this disease, with references to the principal original writers on it, to the article *Spasm of the Glottis*, by Dr. Joy, in the Cyclopædia of Practical Medicine. Dr. Joy, however, takes no notice of the *Thymic* theory of the disease, and refers neither to Mr. Hood nor Dr. Kopp.]—*Hufeland und Osann's Journ. der Prakt. Heilk., Jul.* 1835.—*Ib.*

5. *New Mode of administering Balsam of Copaiba.* By. M. Ratier.—M. Ratier is now in the constant habit of prescribing balsam of copaiba for gonorrhœa, enclosed in small capsules made of gelatin. They each contain eighteen grains of the balsam, and are sufficiently thick to prevent their breaking in the mouth, or giving a disagreeable taste, and yet readily soluble; whilst, from their small size and oval shape, they are swallowed with facility. M. Ratier generally directs half the number prescribed daily for internal use, to be introduced into the rectum, which is easily done if they are covered with oil or suet. As mentioned in our last number, M. R. has been long in the habit of directing his patients to throw into the rectum, by means of a small syringe, one or two teaspoonfuls of the copaiba emulsion, at the same time as he ordered it to be taken by the mouth; but these capsules are in all respects preferable. They were invented by MM. Dublanc, Sr., and Mothes, and are formed by a particular instrument.—*Bulletin General de Therapeutique, Decembre*, 1835.—*Ib.*

6. *On the Effects of Indigo in Epilepsy and other Spasmodic Affections.* By Dr. Roth, of Mayence.—A Number of the *Salzburg Med. Chir. Zeitung*, for 1832, contains a paper on this subject by Prof. v. Leuhossek, wherein it is stated that Prof. v. Stahly, of Buda, had been the first to administer indigo in spasmodic affections, and that the successful results had been noticed in an inaugural dissertation on epilepsy, which appeared at Buda in 1832. This was followed in 1833 by an article in the "Med. Zeitung" by Dr. Grosheim of Berlin, who, it appeared, had employed indigo with entire success in an epileptic case which had previously resisted every other kind of treatment.

These precedents were the occasion of a series of trials being instituted at the Charite at Berlin, by Dr. Ideler, superintending physician of the wards for spasm and insanity, and by Prof. Wolff, chief physician to the other medical wards. as well as by Dr. Leinveber, one of the staff physicians at the Charite.

Although in the selection of cases attention was paid to the remark of Leuhossek that indigo only acted beneficially in the epilepsy directly depending upon abnormal sensibility and reaction of the nervous system, (abnormer sensibilitat und reaction,) the remedy was nevertheless tried on a considerable number of patients; it being frequently difficult to decide whether epilepsy is idiopathic or merely symptomatic, and it also often happening that cases originating as the latter degenerate into the former. At the period when these experiments were made, the greater number of epileptic cases at the Charite differed from each other only in their degree of intensity, and the seat of the affection appeared to be either the brain itself or the cerebral nerves. In no instance did the spinal cord seem to be the chief seat of the morbid action. Most of the patients had already gone through the ordeal of the most powerful remedies without material benefit. At the same time subjects whose epileptic attacks were the consequence of external injury of the head, such as contusions, etc., were not excluded from the trials.

Dr. Roth had an opportunity of witnessing the experiments during a period of eight months, and the following are the general results obtained by him:—The remedy is beneficial in all cases of true *idiopathic* epilepsy, curing those of recent origin and improving [i. e. modifying both the violence and the frequency of the paroxysms] those of long standing. On the other hand few cases of *symptomatic* epilepsy were ameliorated—none cured,—by this treatment.

*Of the Effects of Indigo in the System.* Prof. v. Stahly appears to have been led to the administration of indigo from having observed that the hands of *sickly* indigo-dyers were permanently tinged with blue, which was not the case with dyers in full health. During the first days of its employment, says Dr. G. v. Stahly, jun.,* "the patients are affected with a slight diarrhœa which however ceases spontaneously

*In the dissertation already alluded to.

when the indigo begins to be more easily assimilated with the digestive organs. Even from very large doses, to the amount of two ounces, no other visible effect is produced beyond that of the urine and perspiration assuming a blue color." Dr. Roth however affirms, that with the greater number of patients very different phænomena are observable. In most instances a sense of constriction at the fauces and the impression of a metallic taste on the tongue shortly succeed the first administration of indigo, although the medicine itself is perfectly tasteless. Nausea, inclination to vomit, etc., with delicate persons even vomiting, follow in succession; at times the retching is so violent as to preclude the further use of the remedy, whilst in the other cases the nausea continues for two or three days, and then gradually subsides. The retching and vomiting produced by indigo differs somewhat from those which are the effect of common emetics; the contractions of the abdominal muscles and of the diaphragm being less violent, and the act not being succeeded by the usual feeling of distress and prostration. The egesta present no peculiarity except in their blue color. After all inclination to vomit has ceased, a diarrhœa usually takes its place, increases rather than diminishes as the treatment proceeds, and lasts during the whole time that the indigo continues to be administered. Thus from four to six stools of a semifluid consistency and a blue-black color are produced daily, with the accompaniment of slight gastro-enteric pains, which are likewise observed during the period of nausea and retching, and are in rare instances so violent as to proscribe the further use of the remedy. The protracted diarrhœa ultimately brings on dyspeptic symptoms with vertigo, etc. In one or two instances, the bowels remained obstinately constipated instead of being relaxed. Indigo does not appear to increase the quantity of the urine, but invariably to tinge the latter of a dark violet hue, paler in the urine secreted at night than in that of the morning.

Dr. Roth has never found that indigo affected the color of the perspiratory matter. Slight spasm and twitchings of the tendons sometimes arose after the treatment had lasted for a few weeks.

*Form of Exhibition.* The experiments were made with the Guatimala Indigo of commerce [from the Indigofera Disperma, or the Argentea.] The best mode of administering it is in the form of an electuary, in which one portion of indigo is combined with two of syrup and a small proportion of water. In the form of powder it is apt to occasion distressing spasm at the isthmus faucium. Its tendency to produce diarrhœa may be corrected by the simultaneous use of mild tonics or astringents. The pulv. ipecacuanhæ comp. was found to answer this purpose sufficiently well.

*Dose.* Small doses appear to be wholly ineffectual. If the remedy agrees with a patient at all, it is necessary to commence with tolerably large doses, which may be gradually augmented to an ounce or even more, according to circumstances, per diem; the corrigents to be increased in the same proportion. The treatment it may be necessary to continue for three or even more months.

*Effect on Epileptic Patients at the Charite.* At the commencement of the treatment, the frequency of the paroxysms was in every instance manifestly increased. Each attack was of shorter duration than formerly, but more violent; the subsequent prostration therefore more considerable. The premonitory and soporous stages were less prolonged than usually.

After from one to eight weeks, according to the dose administered, all the above phenomena gradually began to abate. In the successful cases the paroxysms diminished in frequency, duration, and violence, and the last attacks amounted to no more than slight shiverings and inconsiderable twitchings.

The total number of cases of epilepsy treated with the indigo was twenty-six. It has already been stated that only those were cured by it whose epilepsy was idiopathic. The number of cures witnessed by Dr. Roth amounted to nine males and five females. Eleven others were relieved, whilst in six cases the complaint underwent no improvement. Dr. R. was particularly struck with the instance of a boy, aged sixteen, who for eight years had been the victim of chorea St. Viti, to which epilepsy had supervened, but who was radically cured of this double affection by a six weeks' use of indigo.—*Heckers' Neue Annalen. Erster Band. Erstes Heft.* Berlin, 1835.—*Ib.*

7. *On the Relations between the State of the Pulse, Respiration, and Temperature of the Body, in Diseases.* By Dr. Al. Donne.—[The want of accurate information on these points led Dr. Donne to direct his attention to them, whilst acting as "Interne" at the hospitals of la Pitie and la Charite; and, as he has no longer the same opportunities of pursuing his investigations, he has arranged the facts he has collected in tables. Although the inferences he draws from them are but few, and the data not sufficiently extensive, he hopes that he may be the means of directing the attention of others to the same subject. The temperature was taken by a small thermometer placed in the armpit, and kept there for five or ten minutes. To have placed it in the mouth of anus would have been impracticable, and, on a comparative trial of the heat of the mouth and armpit, no marked difference was detected.]

| | Pulse. | Respiration. | Temperature.* |
|---|---|---|---|
| Hypertrophy of the heart | 150 | 34 | 103 |
| Puerperal fever (Metritis) | 168 | 48 | 104 |
| Phthisis | 140 | 62 | 102 |
| Typhoid fever | 136 | 50 | 104 |
| Jaundice | 36 | 37 | 96.40 |
| Hypertrophy of the heart | ... | ... | 94.40 |
| Diabetes | ... | ... | 96.40 |
| Lumbago | ... | 16 | ... |

The preceding cases are those in which the temperature observed was the highest and lowest, and the respiration and pulse most frequent and slowest. It will be observed that, in some of the cases, the temperature was in relation to the rapidity of the pulse and the respiration; but the relations of these three conditions to each other are far from being exact.

*Fahrenheit's scale is used throughout this paper.

### *Pulmonary Tubercles.*

*Women.*

| | Pulse. | Temperature. |
|---|---|---|
| First case | 120 | 102.10 |
| | 128 | 99.50 |
| | 120 | 102-10 |
| | 112 | 100.20 |
| | 136 | 99.50 |
| | 126 | 99.20 |
| | 130 | 100.20 |
| | 140 | 102.10 |
| Second case | 82 | 99.50 |
| | 76 | 99.50 |
| | 84 | 98.30 |
| Third case | 88 | 102.10 |
| | 96 | 100.20 |
| | 76 | 101.30 |
| | 106 | 101.30 |
| | 98 | 100.20 |
| | 98 | 99.50 |
| Fourth case | 100 | 101.30 |
| | 104 | 100.20 |
| | 110 | 102.10 |
| Fifth case | 128 | 101.75 |
| | 120 | 101.75 |
| | 112 | 100.85 |
| | 100 | 100.20 |
| | 84 | 98.30 |
| Sixth case | 68 | 96 |
| | 92 | 103 |
| | 74 | 98.30 |
| *Men.* | | |
| Seventh case, | 126 | 99.50 |
| | 116 | 98 |
| Eighth case | 87 | 99.50 |
| | 62 | 98.30 |
| | 72 | 100.20 |
| | 80 | 101.30 |
| | 88 | 102.10 |
| | 78 | 100 |
| Ninth case | 104 | 101.50 |
| | 104 | 100 |
| | 100 | 100.20 |
| | 110 | 100.20 |
| Tenth case | 76 | 99.50 |
| | 80 | 97.30 |

In some of these cases there is a correspondence between the state of the pulse and temperature. There is not, indeed, an exact correspondence between the number of pulsations and the degrees of the thermometer; but the heat of the body augments at the same time as the pulse quickens, and *vice versa.* This occurred in Cases 5, 6, 7, and 8; but, in Cases 1, 2, 3, 4, 9, and 10, there is no relation; for in some the temperature diminishes when the number of pulsations increases, and in others the heat increases whilst the pulse becomes slower.

### *Pleurisy.*

*Men.*

| | Pulse. | Temperature. |
|---|---|---|
| First case | 106 | 99 |
| | 104 | 100.20 |
| | 106 | 100.20 |
| Second case | 80 | 94.38 |
| | 80 | 100 |
| Third case | 88 | 98 |
| | 80 | 99 |
| | 94 | 98.30 |
| Fourth case | 100 | 101.30 |
| | 100 | 102.10 |

### *Pneumonia.*

| | Pulse. | Temperature. |
|---|---|---|
| First case (*Woman.*) | 102 | 101.30 |
| | 90 | 99.50 |
| | 94 | 100.20 |
| | 98 | 101.30 |
| | 86 | 101.30 |
| Second case (*Man.*) | 92 | 96.40 |
| | 126 | 103 |

### *Hyperthrophy of the Heart.*

*Women.*

| | Pulse. | Temperature. | | Pulse. | Temperature. |
|---|---|---|---|---|---|
| First case | 61 | 97.75 | Fourth case | 106 | 99.50 |
| | 68 | 98.30 | | 108 | 102.10 |
| Second case | 120 | 100 | | 106 | 101.58 |
| | 120 | 101 | | 108 | 99.72 |
| Third case | 64 | 98.30 | | 104 | 99.50 |
| | 98 | 99.50 | | 150 | 103.10 |

### *Chlorosis.*

| | Pulse. | Temperature. | | Pulse. | Temperature. |
|---|---|---|---|---|---|
| First case | 92 | 100,02 | | 100 | 98.30 |
| | 80 | 97.75 | | 82 | 98.30 |
| | 74 | 96.40 | | 96 | 99.72 |
| | 74 | 99 | Third case | 104 | 99.50 |
| Second case | 72 | 99 | | 102 | 100.20 |
| | 96 | 99 | Fourth case | 108 | 100.20 |
| | 100 | 99.50 | | 100 | 101.75 |

### *Metro-peritonitis (Puerperal Fever.)*

| | Pulse. | Temperature. | | Pulse. | Temperature. |
|---|---|---|---|---|---|
| First case | 152 | 102.10 | | 157 | 103.55 |
| | 156 | 102.10 | | 166 | 103.10 |
| | 168 | 104 | | 152 | 102.10 |
| | 152 | 103 | | | |
| | 154 | 103.55 | Second case | 96 | 99.50 |
| | 152 | 103.55 | | 104 | 100 |

### *Jaundice.*

| | Pulse. | Temperature. | | Pulse. | Temperature. |
|---|---|---|---|---|---|
| First case | 36 | 98 | Second case | 62 | 97.75 |
| (*Man.*) | 38 | 98.30 | (*Female.*) | 52 | 96.40 |

### *Hemiplegia.*

| Pulse. | Temperature. | Pulse. | Temperature. |
|---|---|---|---|
| 124 | 99.72 | 96 | 99.50 |

### *Hysteria.*

| Pulse. | Temperature. | Pulse. | Temperature. |
|---|---|---|---|
| 76 | 99.19 | 94 | 98 |
| 94 | 98 | 102 | 99.72 |

### *Diabetes.*

| | Pulse. | Temperature. | Pulse. | Temperature. |
|---|---|---|---|---|
| (*Man.*) | 78 | 97.25 | 84 | 96.40 |
| | 78 | 97.25 | | |

### *Enteritis.*

| | | Pulse. | Temperature. |
|---|---|---|---|
| (*Man.*) | Before bleeding | 104 | 101.58 |
| | After bleeding | 76 | 100.20 |

*Rheumatism.*

| | Pulse. | Temperature. | | Pulse. | Temperature. |
|---|---|---|---|---|---|
| First case ··· | 86 | 98.30 | Third case ··· | 76 | 98.30 |
| (*Man.*) | 82 | 98.30 | (*Man.*) | 60 | 97.39 |
| Second case ··· | 98 | 101.75 | | | |
| (*Woman.*) | 80 | 99.50 | | | |

*Inflammatory Fever.*

| | Pulse. | Temperature. | | Pulse. | Temperature. |
|---|---|---|---|---|---|
| First case ··· | 102 | 104 | Second case ··· | 72 | 97.75 |
| | 72 | 97.75 | | 88 | 101.30 |
| | 60 | 96.40 | | | |

*Typhoid Fever.*

| | Pulse. | Temperature. | | Pulse. | Temperature. |
|---|---|---|---|---|---|
| First case ··· | 88 | 101.30 | Second case··· | 102 | 101.30 |
| (*Man.*) | 90 | 102.10 | (*Woman.*) | 108 | 103.55 |
| | 84 | 101.30 | | 136 | 104 |

From the foregoing observations it appears that a relation between the temperature and pulse existed more frequently than the contrary, especially in those diseases which did not interfere considerably with the function of hematosis. Thus, in tubercular consumption, pleurisy, and chlorosis, there was a more frequent want of relation than there was relation; in organic diseases of the heart, and contraction of its orifices, the relation was by no means constant; in hemiplegia, enteritis, rheumatism, jaundice, inflammatory, intermittent, typhoid, and puerperal fever, the relation was more constant. The constancy in pneumonia was an exception to the other pulmonary diseases.

[Two tables of the state of the pulse and respiration, and of the respiration and temperature, conclude this paper. M. Donne avoids drawing any inferences from these, or attaching much importance to his other deductions, as his facts are not sufficiently numerous; as far however as they go, they are of importance; and, if M. Donne succeeds in inducing others to take similar pains, sufficient data may be eventually obtained to elucidate that very obscure subject, the production of heat.]—*Archives generales de Medicine, Octobre,* 1835. Tome lx.—*Ib.*

8. *Report on Vaccination in France during* 1833. By M. Gerardin.—(*Experiments on the Nature and Reproduction of the Vaccine Virus.*) The numerous cases of modified small-pox which occurred in 1825 in France, in individuals regularly vaccinated, led to the supposition that vaccine matter had degenerated by successive transmissions for a long period. M. Fiard, by a long course of careful experiments, endeavored to settle this point by ascertaining whether or not vaccine virus could now be transmitted from man to cows, and again from cows to man, as it could be at the commencement of this century when it was first introduced into France, as is proved by a report on the state of vaccination, made at that time. If this was not now possible the inference was legitimate that a change in the nature of the virus had taken place. In 1824 and 1825 M. Fiard introduced vaccine matter into seventy cows of different kinds, in only seven animals were pustules produced; the eruption was less developed than in the most feeble vaccinations, and the matter from the eruption introduced into children produced no effect. In 1828 M. Fiard received from England four glasses charged with vaccine matter taken from cows; with this he immediately vaccinated a young cow and the regular eruption followed. He then vaccinated eight children, and in two only was it unsuccessful, one of these having been vaccinated several times with ordinary matter. The eruption differed from common cowpox in the greater severity of both local and general symptoms. Altogether he vaccinated twenty-four children successfully from this same cow. The cow-pox of the inoculated cows was not attended as in the natural disease, as described in this country, by fever, loss of appetite, depression, and diminished secretion of milk. Eventually, from the difficulties attending the transmission of the virus, M. Fiard lost his cow-pox matter. His next endeavors were to ascertain if cow-pox existed among cows in France. He was then living in the department of Ain, and he was assured by the country people that such a disease was common among cows, rendering milking difficult, and that the women who milked the diseased animals always washed their hands in order that they should not communicate the infection to healthy cows. A cow was soon brought to him with several pustules like those of cow-pox on the teats. He inoculated five children with matter taken from these pustules without effect. Twelve days afterwards, on examining the same cow, he found new pustules springing up as the old ones dried up. These circumstances made him doubt the nature of the eruption. Similar experiments were repeated in this district many times in three successive years. In 1827 he repeated them near Paris, and at La Chapelle in 1828, with the same want of success; and he concluded that "it was impossible to procure the cow-pox in France." The information he received from other parts of England confirms a statement of Dr. Baron to M. Valentin as to the rareness of cow-pox in Gloucestershire; during fifteen years it has only appeared at Berkley in 1818, 1819.

[M. Fiard is by no means justified in inferring from his own researches that cow-pox matter cannot be procured in France. His search was continued only a few years, and in two or three districts. As it appeared in Berkley, a village in England, only twice in fifteen years M. Fiard's experiments are certainly too few to admit his sweeping conclusion.*]

M. Fiard instituted the following experiments in order to determine whether (as Jenner thought) vaccine matter came from the disease of horses called, in France, *water in the legs*, in England, *grease;* or whether cow-pox is small-pox communicated to cows; or, finally, if cow-pox is natural to the cow.

*Experiment* 1. In January 1832 M. Fiard inoculated four cows from the matter obtained from the legs of a diseased horse; no pustules were produced.

*Experiment* 2. In January 1833 he inoculated seven cows on their

---

* This criticism is amply justified by a notice, which appeared since it was written, in a French journal, (*Gazette Med.* 2 Avril. 1836,) acquainting us with the fact that the real cow-pox had been discovered at Passy.—Rev.

teats with small-pox matter from a confluent case, without even producing inflammation of the punctures.

*Experiment* 3. In September 1833 he inoculated seven other cows with confluent small-pox matter without effect.

About this period the following experiments were made at the veterinary school at Alfort, to determine the value of the statements of Dr. Sunderland. Their object was to ascertain if small-pox could be communicated to cows by exposing them to the contact of the body and bed linen of those who had suffered from the disease. After having ascertained that there were no pustules of cow-pox on three cows, two of the animals were covered by a sheet and counterpane, and a third with a sheet, which had been used by a small-pox patient at the Hotel Dieu, and on which were observed stains made by the dried virus. These were fixed by belts to their bodies and remained on ten days and ten nights. During this time and twenty days afterwards the cows were seen every morning, but no trace of the disease was detected. Other pieces of linen were applied for seventeen days, and the teats rubbed with a shirt impregnated with virus, but no symptoms appeared though the cows were watched for the next month. In order to ascertain if the linen actually possessed contagious properties, the bodies of a dog and of a pig were each surrounded with one of the impregnated napkins which had not been used in the previous experiment; as each of these animals are known to contract the disease. The dog released himself from the napkin in twenty-four hours; the pig in ten minutes; and it was torn in pieces by other pigs with him. The two animals were examined every day for a month, and nothing appeared. Twenty-three days after the experiment a pig was observed to have a crop of pustules, like the natural small-pox in these animals, on the skin of his testicles, belly, and thighs; and eight days afterwards other pigs had a similar eruption, which ran through its regular stages. From these experiments at Alfort it appears. 1. That the small-pox could not be communicated to three cows by simple contact. 2. That it is probable the three pigs which destroyed with their teeth the napkin stained with virus, contracted the small-pox from the human subject. At the same time similar experiments were made at Rambouillet by Dr. Brunelle on cows and on four sheep; these are detailed. but the plan adopted was much the same as at Alfort, though more care was taken in procuring fresh small-pox virus. The animals contracted no disease.

Two individuals died of small-pox in a sort of cellar badly aired, and communicating with another cellar in which two cows were kept which were obliged to pass through the place where the sick persons lay. Although the clothes, etc., of the deceased persons remained more than fifteen days in the cows' stable, the animals were unaffected. A man lay ill of the small-pox in a stable with three cows who were not unaffected. Two setons steeped in the pus of small-pox were inserted into a young cow without inoculating her with the disease.

[Notwithstanding these results, the Academy does not regard the question as settled, but recommends it to further attention. The method by which cow-pox could be reproduced at will, would probably open a new field for research; and complete the benefits of Jenner's discovery.]—*La Lancette Francaise. 2 and 5 Mai*, 1835.—*Ib.*

9. *On the Treatment of Inflammation of the Testicles by means of Compression.* By J. C. F. Fricke, Surgeon to the General Hospital in Hamburg.—[In presenting to our readers the following important document relative to a new mode of treating Hernia humoralis, or swelled testicle, we shall for the most part make use of the author's own words, merely omitting phrases and brief paragraphs, here and there, which do not seem essential. It may be well to inform the reader, that Dr. Fricke is a surgeon of great reputation and of most extensive experience, and the author of some surgical works of much practical value.

I had long meditated (says Dr. Fricke,) on the discovery of some means to obviate the tediousness and other numerous inconveniences at ending the common mode of treating inflammatory affection of the testicle, by leeches, poultices, etc.; and at length it occurred to me that *compression*, which I had found so very serviceable in some analogous cases, offered the fairest prospect of a favorable result. The event completely answered my expectations; and I had soon the pleasure to perceive how, by means of this, the disease could be removed, in a simple, easy, and surprisingly rapid way.

Generally speaking, compression may be employed in every kind of inflammatory enlargement of the testicle, and from whatever cause produced. We have found it equally useful in cases arising from gonorrhœa, whether springing from sympathy in the inflammatory stage, or originating in what is called suppressed claps, and in such as have arisen from external injuries. The degree or period of the inflammation makes no difference.

The only contra-indication to the employment of this treatment, worthy of consideration, has been found in an affection of the general system. For instance, if the local inflammation had arisen from errors in diet, such as abuse of spiritous liquors, or if, contemporaneously with it, considerable disorder of the gastric system had shown itself, it was found necessary to remove this state before recourse was had to compression; as, otherwise, the usual result was not obtained, and the employment of compression was obliged to be postponed for a period.

In many cases the compression at first increased, in some degree, the pain of the inflamed testicle; in some cases (particularly when applied too forcibly,) it produced great pain; but this never continued long; the patient, after a short time, often in a quarter of an hour, and even in cases where the pain had been extremely severe, finding himself so completely relieved as to be able to leave his bed and to walk about in his room.

In inflammatory swellings of quite recent origin, a single application of the compression was found sufficient, in many cases, to remove the disease. When it was of longer duration, (say, from three to eight days,) it was found necessary to repeat the compression two or three times. Swelling of the spermatic cord, if it was not very considerable, did not at all contra-indicate compression; nor yet did other contemporaneous local affections, such as buboes, ulcers, etc. When a general febrile state was produced by the orchitis, compression was found the best means speedily to remove it, at least where the vascular reaction was not too great; although, in extremely rare cases, this was produced by the compression itself.

The unpleasant part of the treatment by compression was, as I have said, its occasioning pain in some cases. This result was observed chiefly in the early period of my practice, and I considered it as owing to our making the compression too strong. In my latter practice, on avoiding this, we heard no more of pain being produced by it. In some cases, in which the affection had been previously treated by cataplasms, etc., and where we had only made one application of the compression, there still remained for some time a slight painful swelling of the testicle; but it gradually disappeared.

In several cases I had occasion to observe, as the consequence of compression, nausea, inclination to vomit, and bitter taste in the mouth, coming on without any other evident mark of gastric disorder. When this was the case, compression evidently was of no avail; the pain remitted little or not at all, and the swelling did not decrease. On removing the compression, giving an emetic, or applying a poultice to the stomach, the symptoms of disturbance soon disappeared. In the few instances in which this affection of the stomach was observed, the compression had been for the most part too strong; in two of them, however, it seemed to depend on previous disease in the abdomen. It is however to be observed, that the cases in which this sympathy exists in such a degree as to give occasion to gastric disorder are, generally speaking, so rare as not to be regarded as any drawback on the superiority of this mode of treatment. It is necessary, however, in all cases where such a disposition shows itself, immediately to put an end to the compression.

The good effects of the compression show themselves very soon after its application, and the speedy abatement of the pain is always the surest sign of its efficacy. If the pain continues some hours in any considerable degree, a general disorder of the system may be looked to as explaining the failure of the treatment.

I will now give a comparative statement of the results of the treatment of orchitis by leeches, cataplasms, etc., and of that by compression, taken from the journals of the General Hospital, since the commencement of the practice in 1832. In all, we have compared seventy-four cases: of this number, fifty-one may be regarded as acute cases, or cases in which the symptoms of inflammation were strongly marked, and twenty-three as chronic cases, or cases in which the inflammatory symptoms had more or less remitted. Of the first division (of fifty-one), eighteen were treated with leeches, cataplasms, etc., and thirty-three by compression;—of the second division (of twenty-three,) nine were treated with poultices, leeches, etc., and fourteen by compression. The following are the results of the two different kinds of treatment, as regards the time occupied during the case:—Of the thirty-three cases of acute orchitis treated by compression, the average period of treatment was nine days;—of the eighteen acute cases treated without compression, the average was thirteen days:—of the fourteen chronic cases where compression was employed, the average period of treatment was twelve days;—of the nine cases submitted to other treatment, the average was fourteen days. Such were the average results; some of the comparative results of the two kinds of treatment, in reference to individual cases, were as follows:—Of the thirty-three acute cases treated by compression, five were cured in three days; five, in five or six days; six, in seven days;—of the eighteen acute cases treated by other means, one case was cured in three days; one, in five days; two, in from seven to eight days, seven, in from eight to eleven days. In regard to the chronic cases, out of the fourteen treated by compression, one was cured in two days, and the greater number in ten or twelve days; while, of the nine cases in which cataplasms, leeches, etc., were used, the cure took place in no case in less than eight days.

Latterly, when experience had enabled me to treat the disease with more circumspection, the results of compression was much more favorable. In the present summer (1835,) I treated in this way seventeen cases, which are not included in the above statement. Of these were cured in one day, one; in two days, four; in three days, four; in four days, two; in five days, three; in nine days, one; and two in ten days. The three last were severe and unfavorable cases. In nearly two-thirds of the whole of the above-mentioned cases, no hardness or swelling of the testicle remained behind.

I will now describe the manner in which I apply compression. At first I attempted to compress the testicles against the thigh and pelvis, by passing over it long and wide strips of sticking plaster, from the nates up to the abdomen. I was soon forced to give up this plan, as well because the compression produced by it was neither secure nor equal, and the patient was forced to keep himself in bed, and even while there, to avoid all considerable movements. After many other unsuccessful attempts by means of temporary bandages, etc., I at length adopted the following, which is proved by experience to be the best.

For the purpose of compression, I employ strips of sticking plaster; the plaster being made very adhesive, but not of too irritating materials,* and spread on linen the breadth of the thumb. No preparatory measures are required, no leeches, cataplasms, etc.

In slighter cases the patient may stand before the surgeon, leaning against the wall, or he may rest on the edge of the bed or sofa, in such wise that the scrotum may hang freely down. If the scrotum and neighboring parts are much covered with hair, this must be removed: but, generally speaking, this is unnecessary.

The surgeon takes the scrotum in one hand, and separates the diseased from the sound testicle; while with the other hand he gently stretches the skin of the scrotum over the former; the spermatic cord is isolated in the same manner. If the testicle is much swollen, it must be held by an assistant; otherwise, it suffices for the patient himself to keep the sound testicle somewhat separate from the diseased. The surgeon now applies the first strip of plaster over the isolated spermatic cord, about a finger's breadth above the testicle,

---

*The following composition, contained in our Codex, is the plaster which I have employed for some years, and has been found preferable to all others:

R. Emplastri Lithargyri, partes sex;

Colophonii pulverati (Picis nigræ,) partem unam. Seorsim liquata commisceantur.

The Emplastrum Lithargyri employed is made as follows:

R. Lithargyri subtilissime lævigati, lib. v.
Olei Oliv. lib. ix. M. Coque igne moderato, spatu'a lignea semper agitando et pauxillum aquæ subinde instillando, donec Lithargyrum perfecte solutum sit, etc.

holding the end of the strip with his thumb, and passing it round the cord. He proceeds in the same way with the second strip, which must either in part or altogether cover the former. The first part of the process must be carefully done; the strips must compress the cord closely, (and for this purpose it must be kept approximated to the skin, which is to be tightly stretched over it;) otherwise, when the other extremity of the testicle is compressed, the upper end will be apt to slip upwards through the loose rings of sticking plaster; a circumstance not only occasioning pain, but rendering the whole operation abortive. In this manner we proceed, laying strip after strip, the last always lying over the former by a third of its width, until we have reached the thickest part of the testicle, and where it begins rapidly to decrease in diameter. The surgeon now changes his mode of proceeding, and, laying hold of the testicle already covered, passes his strips from above downwards over the lower portion of the testicle, and up over the back part. In this way the whole remaining portion of the testicle is closely enveloped and compressed. I have already said that the compression must not be too great; and in most cases the surgeon will be ablo to judge as to the proper degree by the speedy disappearance of all the pain which had previously existed.

If both testicles are affected, we proceed to envelope one in the manner now described, when this is done, it will be found that there is not room left for applying the circular strips in the same manner to the other; we are therefore under the necessity of including both testicles in the circular strapping, the testicle already covered serving as a point of support for the other. Over the lower portion of this second testicle the strips are passed, as in the former case, from behind backwards.

In some cases where the skin is irritable, ulcerations take place; in this case small slits must be cut in the plaster, and a goulard lotion applied, which soon heals them.

Generally speaking, the patients can leave the bed immediately after the strapping, and walk about the room; and, in cases where the inflammation is not very great, or has been taken early, they may even go out and work a little.

The renewal of the straps must depend on the decrease of the swelling and other symptoms. In many cases one application suffices; otherwise, we remove the plasters when they have become so loose as to admit the introduction of the scissors between them and the skin.

Any other treatment the patient may require must depend on the complications of the disease; the orchitis, as such, needs nothing besides the compression.

In those inflammations of the testicle which originate from blows or pressure, etc., compression has proved the best treatment. Here, if the inflammation ran very high, I have usually applied leeches in the first instance, and kept on poultices for one or two days; but in slighter cases I had recourse immediately to compression.

The following are the principal advantages which the treatment of orchitis by compression possesses over other methods:

1. The speedy removal of pain.
2. The quick removal of the disease itself.

3. The simplicity of the method, and the slight trouble thereby given to the patient.

4. The small expense of the treatment.

5. The comparatively slight care and attendance required on the part of the surgeon. The two last points are of considerable importance in hospital practice.—*Zeitschrift fur die gesammte Medicin.* B. i. h. I. 1836. *Hamburg.—Ib.*

10. *On Suicide in the Canton of Geneva.* By M. Prevost.—[By the laws of the canton, each case of violent death is investigated by a police magistrate, and the documents are sent to the "procureur generale," and carefully preserved. M. Prevost has examined these documents, collected between 1825 and 1834 inclusively, with a view to investigating the causes of suicide, and of diminishing them, if possible. The following are the more important results.]

1. *Age.*

| Ages. | Number of cases in ten years. | Men. | Women. |
|---|---|---|---|
| From 50 to 60 | 34 | 25 | 9 |
| 20 to 30 | 30 | 22 | 8 |
| 60 to 70 | 19 | 10 | 9 |
| 30 to 40 | 18 | 15 | 3 |
| 40 to 50 | 15 | 13 | 2 |
| 70 to 80 | 9 | 6 | 3 |
| 10 to 20 | 5 | 3 | 2 |
| 80 to 90 | 3 | 1 | 2 |

From this table it appears that suicides are most frequent between 50 and 60 years of age. The age when the passions are the strongest (from 20 to 30) is, as might be expected, high in the scale; that of youth and old age low, from the young being strangers to the cares of life, and the old few in number when compared with the population.

2. *Sex, and State of Marriage or Celibacy.*

There are more suicides among men than women, in the proportion of 95 to 38, or about three to one; and more unmarried than married, or in the state of widowhood, in the proportion of 70 to 63, or about seven to six. Notwithstanding this, the female suicides are more numerous among the married and widows than among the unmarried, in the proportion of 21 to 17. But among men the proportions are reversed, that is, 42 to 53; so that, on the whole, suicides are more frequent among the unmarried than amongst those who are or have been married. This will not surprise those who know the energy, courage, and patience of women under misfortune; men more readily giving way to despair, and to vices consequent upon it. Men also have means of destruction, as fire-arms, more readily at hand.

3. *Occupations.*

The number of suicides are in proportion to the numbers of the individuals engaged in various trades, except among the agricultural population, where the proportion is very small. Thus, the agricultural population of the canton is 18,000, among whom, during ten years, there have been but twelve suidices; whereas, if they had been in the same proportion to the whole number as was found in the other occupations, they would have amounted to thirty-nine. Constant occupation, and hard yet, healthy work, render them less sensible to the cares of life. There is also a somewhat larger proportion of suicides among the educated classes, who are engaged in literary pursuits or the higher branches of commerce.

4. *Religion.*

The relative proportion of Protestants to Catholics in the canton of Geneva is, according to the census of 1834, as 77 to 56. Thus,

| Of 133 inhabitants, there are | | Of 133 cases of suicide, |
|---|---|---|
| Protestants | 77 | 107 |
| Catholics | 56 | 26 |
| | 133 | 133 |

This result should attract the attention of those who are interested in the moral and religious education of Protestants.

5. *Means of Destruction.*

| | |
|---|---|
| Drowning | 55 |
| Fire-arms | 31 |
| Strangulation | 18 |
| Voluntary falls | 15 |
| Cutting instruments | 7 |
| Poison | 7 |
| | 133 |

In a small province with a lake and two rapid rivers, it is not surprising that drowning should be the most frequent mode of suicide; next to this is death by fire-arms, which is accounted for by all the men having fire-arms, as they are in the militia. Whilst the men have used fire-arms and cutting instruments, the women have almost alone had recourse to poisons and voluntary falls.

6. *Seasons.*

The seasons influence sensibly the number of suicides. There are more almost constantly in April.

Of 133 suicides, there were in

| | | | |
|---|---|---|---|
| April | 19 | March | 10 |
| June | 17 | November | 9 |
| August | 17 | September | 6 |
| July | 15 | January | 5 |
| October | 14 | February | 5 |
| May | 13 | December | 3 |

The spring appears to have an unfavorable effect, and during the great heats there are more suicides than during the cold weather. It is curious that many suicides happened on the same day or week. Thus, on April 9th, 1830 there were two suicides, and several others on the previous and subsequent days; on the 20th May, 1830, there were two suicides; on the 28th and 29th of March of 1831, two; and the same on the 3d and 4th of July of the same year. On April 20, 1833, there were two, and, on July 5th, 1833, two others. Some atmospheric changes may account for this, though meteorological tables did not satisfactorily explain them.

7. *Presumed Motives.*

| | | | |
|---|---|---|---|
| Physical disease | 34 | Bad conduct. Drunkenness | 10 |
| Insanity | 24 | Fear of punishment. Remorse | 6 |
| Losses of property | 19 | Disappointment in love | 6 |
| Domestic grief | 15 | Gambling | 4 |
| Melancholy without known cause | 13 | Mysticism | 2 |

8. *Relation of Suicides to the Population and to the Deaths.*

The number of suicides is, as to the whole number of deaths, as one to 90½, and to the whole population as one to 3,985; the mean population of the canton during the last ten years being 53,000.

| | | |
|---|---|---|
| In 1825 | 6 | suicides. |
| 1826 | 6 | — |
| 1827 | 9 | — |
| 1828 | 13 | — |
| 1829 | 13 | — |
| 1830 | 16 | — |
| 1831 | 18 | — |
| 1832 | 12 | — |
| 1833 | 24 | — |
| 1834 | 16 | — |
| | 133 | |

From this table it appears that the number of suicides has gradually increased from 6 as high as 24 in one year. The last year it decreased to 16; and it is fervently hoped that this deduction may be maintained, and that the increase may not be so frightfully rapid as it appears to have been. It must, however, be taken into account, that the population was, in 1822, 51,113, and, in 1834, 56,655. The police also are more active, and inquests are held more regularly.

(Extracted from the "Bibliotheque Universelle." *Geneve.* Juin, 1835.) *Annales d'Hygiene publique.* *Janvier*, 1836. No. 29.—*Ib.*

10. *Detection of Sugar in the Blood in Diabetes, and on the best Mode of separating the same substance from the Urine.* By Felice Ambrosioni, of Milan.—[It is well known that until very lately the most distinguished British and foreign chemists have failed to detect the presence of sugar in the blood of diabetic patients. This, however, has been recently effected by the author of the memoir of which we shall give an extract, and also, we believe, by some other continental chemists. If this does not prove to be only an exception to the general rule,—and we confess we are sceptical on the point, on considering that such eminent men as Guendeville, Nicolas, Wollaston, Henry, Marcet, Prout, failed to detect any trace of sugar in their experiments,—the discovery will occasion a great change in our views respecting the pathology of diabetes. It will lead to the conclusion, that in diabetes, as in other diseases, the office of the kidneys is chiefly to act as filters to the blood, by removing effectes and unassimilated matters, also that the disorder does not reside in these organs, and consequently that our remedies must be directed to the improvement of the other functions. The following are Signor Ambrosioni's processes for detecting the saccharine principle in the blood and urine. It is proper to observe, that the discovery was made only in a single case; and it may not be uninteresting to mention that the case was cured by the use of creosote.]

I. *Analysis of the Blood.*

To obtain sugar from the blood, one pound of the serum and crassamentum from the same patient was diluted with water boiled and filtered. The filtered liquor was deprived of its color by subacetate of lead, and a stream of sulphuretted hydrogen threw down from the mixture a black pultaceous mass, which diluted with distilled water, and filtered, produced a dark brown liquor, which was boiled in a solution of albumen. As the albumen coagulated, the liquor separated into a clear colorless fluid and a dark flocculent and insoluble matter. The fluid portion, when boiled slowly, threw up a syrupy scum, and assumed all the characters of a perfect syrup. Left at rest for a few weeks, small colorless crystals were formed in it of a prismatic form, with a rhomboidal base, modified at the angles and apices, in every respect resembling a perfect sugar. The uncrystallized syrup, at about 80° F., mixed with a little yeast, exhibited a decided vinous fermentation. The quantity of syrup obtained from one pound of blood was about an ounce, the crystals weighed two grains. [Since the foregoing was transcribed for the press, a case has been published in the Medical Gazette, No. 431, in which sugar was detected in the blood by Mr. Maitland.]

II. *Analysis of the Urine.*

The process employed in the reduction of sugar from the urine is as follows: The urine, which was pale, void of urea, and contained few salts, was treated with subacetate of lead, the oxyde of which was precipitated, combined with the animal and coloring matters, and a few salts. Sulphuretted hydrogen, passed through the filtered liquor, removed the excess of the salts of lead, which formed a brown precipitate, the liquor remaining clear, and consisting of water, sugar, and a minute portion of animal matter. Evaporated in a water bath to a syrup, and left for some weeks at the ordinary temperature, a white efflorescence beginning at the sides of the vessel, gradually extends over the whole, and converts it into a solid, whitish, amorphous mass, which when broken, exhibits indistinct traces of crystalization; and, when washed with pure alcohol, is freed from the animal and coloring matters, and remains undissolved in the form of a pure white sugar.—*Annali Universali di Medicina. Aprile*, 1835. —*Ib.*

12. *Experiments on the Action of Oxalic Acid.* By J. W. Arnold.—These experiments were performed on dogs, cats, birds, and (chiefly) on rabbits; in the latter, the acid does not produce vomiting, and consequently it is unnecessary to tie the œsophagus. The poison was introduced into the stomach by pouring it slowly into a horn funnel attached to the outer end of an elastic tube passed down the gullet,—a mode of proceeding which makes it unnecessary to confine the animal.

The following are the general conclusions drawn from the experiments made with acid prepared and administered under various circumstances.

1. Oxalic acid is an active poison, and when given in large doses usually proves quickly fatal.

2. Its poisonous effects are uniform, and independent of extraneous admixtures, the mode of preparation, etc.

3. Its immediate effect on the nervous system is stimulant. This primary excitement is more or less rapidly succeeded by diminished vigor of the nervous functions.

4. The effect upon the heart is of the same kind, and dependent upon that produced on the nervous system; it occurs very speedily,—by a very transient state of extraordinary excitement being succeeded by diminution and ultimately by cessation of the heart's action. Similar observations are applicable to the respiratory organs.

5. The blood is peculiarly affected by oxalic acid. After large and speedily fatal doses it is fluid, uncoagulable; which is not the case after smaller and less rapidly destructive doses. This action cannot be ascribed to a chemical solution, as is sufficiently proved by its succeeding only to a quick, almost sudden death from the poison. Further, the coagulum of blood macerated at an elevated temperature, for a month together, in a solution of the acid, is not affected in this manner. It may consequently be assumed that the blood is thus affected by the acid as a result of the action of the latter on the nervous system; a supposition that will not be rejected by those physiologists, at least, who consider the blood as capable of being immediately influenced by the nervous system. The great accumulation of the blood in the venous portions of the heart and vascular system is to be explained by the impaired state of respiration and circulation. Like other acids, as Stevens and Hartwig have shown, the oxalic renders the color of the blood dark.

6. As regards the action on the alimentary canal, it operates as a local irritant, causing inflammation and extravasation of blood,—effects which are less obvious in proportion to the rapidity with which death ensues. Black points and patches occur on the mucus surface in consequence of the chemical action on the effused blood. The solution of the mucus membrane appears also to depend on chemical causes, inasmuch as it is always more decided in proportion to the interval which elapses between death and the time of examination.

7. The best means of counteracting the operation of the poison are those which tend to its evacuation; among which, emetics, however, are the less to be recommended, as the vomiting produced by the poison itself has little efficacy in preventing its injurious consequences. The stomach-pump is the most certain, if it can be used early enough. The employment of such bases as unite to form soluble salts with the acid, is of no service, as such salts are themselves poisonous.—Magnesia and lime, on the contrary, deserve attention, as their combinations with the acid are insoluble. Stimulants, such as alcohol and camphor, have been proposed to counteract the depressing effects, particularly on the nervous system: but the rapidity of a fatal termination from large doses of the poison usually renders all aid useless.

8. Lastly, these experiments prove, what had been already rendered probable by observations on the human subject in health and disease, that neither the dilatation nor contraction of the pupil is to be considered as a simply passive condition.—*Tiedemann's Zeitschrift fur Physiologie.* Vol. V. 1835.—*Ib.*

13. *On the Present State of Medicine in Denmark.* By C. OTTO, M. D., Professor of Medicine in the University of Copenhagen. First report on the Medical Institutions and the Medical Profession. There are two public establishments for medical and surgical instruction in Denmark; the one the *Medical Faculty* at the University, the other the *College of Surgery*.

### A. THE MEDICAL INSTITUTION AT THE UNIVERSITY.

Denmark possesses two universities; one for the German provinces, at Kiel in Holstein; another for the Danish provinces in Copenhagen, on the Island of Zealand. The university of Keil is, in almost all respects as to plan, the period of study, etc., like those in Germany; but that of Copenhagen, although in the beginning principally modelled after its sisters in Germany, differs at present in many respects from them.

### I. THE UNIVERSITY OF COPENHAGEN.

This was founded in the year 1479. The number of the professors, who are divided into the four usual Faculties, amounts at present to thirty-eight; but there are, besides these, one or two private teachers in every one of the Faculties. Some (the oldest) of the Professors are *Ordinary*, others (the younger) *Extraordinary*. The Ordinary Professors have a higher rank, have a seat in the *Consistorium* (the academical senate,) and a better salary. Every year a new rector of the University is chosen, and out of every faculty alternately, but only from among the ordinary professors. The rector has during his office a very high rank, is addressed, "Your Magnificency," and gets a salary of about four hundred Danish dollars. The income granted to the professors is partly derived from domains and farms given to the University at its foundation, partly from rents of churches, and partly from royal and private bequests.

The buildings of the university were for the most part burnt in the bombardment by the English in 1807, and were not rebuilt until the last year. They are now completed with a degree of taste and magnificence far surpassing the old; and the initiation of the restored institution is to take place this year, at the Jubilee of the great reformation of the church, which was introduced into Denmark in the year 1536. Besides the proper buildings of the university, there are four houses (formerly they were eleven, but seven were burnt in the year 1807,) in which four of the eldest professors with their families reside free of expense. The university also possesses funds derived from considerable legacies and stipends, and which are destined for the support and benefit of the students, for the enlargement of the library, for defraying the traveling expenses of promising candidates, etc.

Amongst these are the following: 1. The *Community* and *Regents;* the first is a weekly stipend, and the second is a free lodging for one hundred students at the *College of Regents*, a large edifice, founded by King Christian the Fourth. 2. The *foundation of the College of Walkendorff*, the oldest in the records of Denmark, having been instituted in 1595, by C. Walkendorff, steward of the Empire, for the free residence of sixteen students. 3. *Borch's College*, called also "Collegium Medicum," built and endowed by the learned C. Borch, for the support of sixteen students. 4. *Elersen's College*, founded and endowed in the year 1689, by Jorgen Elersen, counsellor of state, also for the support of sixteen students. Independently of these foundations, many bequests have been granted to the university, the interest of which is appropriated to the support of indigent students and to the general extension of literature.

In order to authorize a student to be matriculated at the university, and to get admittance to the lectures, a public examination must be gone through: this is held once annually (in October,) and is called "examen artium." At this the candidate, recommended by the rector of a preliminary school or by a private teacher, is examined publicly, both by means of written exercises and *vive voce* in every thing, relating to "humaniora," viz: Latin, Greek, history, geography, mathematics, religion, the living languages, etc. The examinations are conducted by the professors in the Philosophical Faculty. It is not allowed to any student to undergo this examination before the age of seventeen; or, at least, a dispensation from this rule can only be granted by a special permission of the *Royal Board of the University*, which will be noticed hereafter. The examination is one of the most impartial and at the same time the most rigorous; and no one who is deficient in the knowledge of the several branches of classical and literary education can obtain a character, (there are three,) which will enable him to procure "testimonium civis academici;" in other words, which will gain him admittance to the university, and confer on him the name of "student;" a name, which in Denmark is more respectable than anywhere else. Matriculation without this "examen artium" is only allowable to those who, after having been matriculated at some other university, intend to continue their studies here or to take the degree of doctor.

As a stimulus to diligence and literary labors, eight prize-essays are annually proposed in eight different branches (theology, law, medicine, philosophy, mathematics, philology, history, and æsthetics,) to which only students and candidates without any public office are allowed to aspire. The prize is a golden medal (on one side of which is the figure of Minerva, and the other a wreath of laurel, with the legend—"Studio et Ingenio,") or the sum of 5*l.*

The number of students at the university of Copenhagen has been increasing for many years; and, considering that these consist of natives only, the fact proves a laudable wish of the Danes in general to give their children a literary education. It is even very common for the sons of tradesmen to be brought up at the university. One of the causes which contribute to keep up the number of students is the comparatively small sum of money required for matriculation at the university (sixteen dollars, or 1*l.* 12*s.*) In order to prove the increasing number of students, I subjoin the following table, by which it will be seen that, even after the year 1811, when Norway got its own university by the bounty of the present king, the number did not diminish very much, and has since been increasing considerably.

| Years. | Students. | Years. | Students. | Years. | Students. |
|---|---|---|---|---|---|
| 1808 | 90 | 1813 | 84 | 1818 | 111 |
| 1809 | 83 | 1814 | 107 | 1819 | 102 |
| 1810 | 83 | 1815 | 98 | 1320 | 139 |
| 1811 | 183 | 1816 | 81 | 1821 | 129 |
| 1812 | 102 | 1817 | 116 | 1822 | 153 |

**etc., etc., etc. At present the number of students is from 700 to 800.**

But whilst there was so considerable a number of students at the university, the proportion of medical students was small, being not more than one or two in the hundred. The chief reasons of this were undoubtedly the greater expenses which medical study involves, and the few public offices in medicine compared with those in law and in theology. But in later years the number of medical students has increased considerably, so that there are now often ten or twelve in every hundred who are matriculated. The natural consequence of this great increase has been an overwhelming number of practitioners, who however clever and active, are not able to get any public employment, and, consequently, only with difficulty and after long struggles are enabled to earn a livelihood. Nevertheless, they continue to form part of a class the most respectable in morals and the most liberal in principles.

### II. THE MEDICAL FACULTY.

In the medical faculty at the university of Copenhagen there are three ordinary and two extraordinary professors, who are appointed by the Royal Board of the University, according to a recommendation of the Faculty, but which choice must be approved of by the king. They all get an annual salary; and from among the ordinary professors there is every year elected in rotation a Dean, who superintends the affairs at the Faculty. Besides these five professors, there may be an unlimited number of private teachers or lecturers, yet not without having taken previously the degree of doctor at the university; otherwise, a special permission is to be obtained. There are at present only two such private teachers, so that the number of lecturers at the Danish Medical Faculty does not amount to more than seven. This is a number which certainly is not at all adequate to the teaching of the numerous branches of medicine; and accordingly, several branches, as for instance dietetics, military hygiene, semeiotics, the history of medicine, morbid anatomy, etc., are not at all taught. This is a deficiency in the Faculty, which we hope will soon be remedied by the union of the medical faculty with the other public establishment for medical and surgical education, of which I shall afterwards speak.

The following are the names of the teachers, and the subjects belonging to their respective chair:

*Ordinary Professors.*

| | |
|---|---|
| J. L. Saxtorph, | Surgery, Midwifery, the Diseases of Women and Children. |
| D. J. Herhholdt,* | Human Physiology. |
| C. L. Bang, | Pathology, Therapeutics, Clinical Medicine. |

*Extraordinary Professors.*

| | |
|---|---|
| D. F. Eschricht, | Anatomy and Human and Comparative Physiology. |
| C. Otto, | Pharmacology and Forensic Medicine. |

*Private Lecturers.*

| | |
|---|---|
| E. Svitzer, | Anatomy and Dissections. |
| S. T. A. W. Stein, | Anatomy. |

* Since dead.

In the Philosophical Faculty of the university are given lectures on the collateral branches of medicine, botany, chemistry, zoology, mineralogy, etc.

It is of course here, as in other universities, that the professors are not restricted to give lectures only in those branches to which they are appointed, but may lecture upon any other they choose. This, nevertheless, very seldom happens, and it will at once be seen on what unequal terms the different branches of medicine stand at the Danish University. Anatomy has three lecturers physiology, two; while semeiotics, morbid anatomy, mental diseases, etc.; have none at all. The same is the case with some other branches of therapeutics, as for instance, diseases of the eyes, of the ears, of the teeth, etc.; an anomaly which also will find a remedy in the above-mentioned union of the two establishments for medical education. Another defect in the Medical Faculty at the university is the very small salary which the five professors receive for their labors; although they lecture almost every day during eight months of the year, the ordinary professors do not receive annually more than 1000 Danish dollars (100*l*.) and the extraordinary ones only 800 (80*l*.) The natural consequence of this is, that the medical professors, as they do not get one farthing from the students, the lectures being all public and gratuitous, in order to obtain a livelihood, are obliged to endeavor to procure some additional office, and also to enter into private practice; the consequence of which is the impossibility of their paying that exclusive attention to their professorships which these require. Yet it is exactly on this false principle, that the medical professors will be able to increase their income by practice, that the Royal Board of the university is acting in appointing a less salary to them for their labors at the university than to the professors of the other faculties; a principle which certainly is to be blamed in the severest terms, inasmuch as the salary should be sufficient to enable the professors to do without practice or any other offices, so as to devote themselves entirely to the duties of their chairs. The professors may certainly besides their public lectures, give private ones, i. e. such as the students must pay for; the sum paid for such private lectures is only five dollars (2*s*. 6*d*.) for each [course?] and even this small sum the medical student is so unaccustomed and so disinclined to pay, that he very rarely attends such lectures. And truly, for this reason, the professor is even ashamed to ask his fee of the few who do attend. The same principle operates also as a cause of the impossibility of the professors occupying themselves with literary labors; for who that is forced to spend the whole day in the labors of an active practice is able to devote the few remaining leisure hours to literary studies? or who that has two offices to attend to (and all the professors of the Medical Faculty have more than one office,) can be expected to have much time left for any scientific occupations, except such as are involved in the discharge of the duties belonging to those offices.

Before he can undergo his examination at the Medical Faculty of the University, the medical student must have gone through the following preliminary trials:—He must be matriculated as student at the university, i. e. he must have taken his examination at the "examen artium." 2. He must furthermore have undergone the *philosophical examination* (examen philosophicum) which commonly takes place in the course of a year after the "examen artium," and is divided into two parts, at the first of which (examen philologicum) the candidate is again examined in Greek, Latin, history, mathametics archæology, and natural history; and at the second (examen philosophicum proprie sic dictum,) in physics, astronomy, and the several branches of philosophy (logic, morals, metaphysics, etc.) The object of these preliminary examinations is to give the examiners a proof of the progress of the student in his literary career, and to oblige him to imbibe a knowledge of the natural sciences, without which he never will become a true, learned, and scientific physician. After these two examinations, the student may apply himself to the study of medicine; and when he requests to be examined at the "examen medicum," he must, 3, in addition to the foregoing, produce certificates of having attended the several lectures of the Professors of the Faculty, and of having visited the hospitals.

There is no period fixed for the study of medicine, as at the Austrian and many other universities; neither are certian lectures appointed to be attended the first, the second, the third year, and so on. Such supererogatory interference certainly only tends to stifle genius, and to make diligence and industry of less avail than they ought to be. It is left entirely to the candidate in how many or in how few years he shall finish his studies, but the usual term is four or five years.

When the candidate is admitted to the "*examen medicum rigorosum*" by the Medical Faculty, he must undergo the following trials of his knowledge:—1. He must examine a patient, newly taken in at the Fredrick's Hospital (the first in town,) explain the symptoms of the case, assign it a name, tell the seat of the disease and its remedies, prescribe the indicated medicine and the diet, etc. 2. He must give, in Latin, written answers to a physiological and a therapeutical question. 3. He must make two anatomical dissections in the dissecting room. 4. He must perform two surgical operations, explain their nature, the different modes of proceeding, the possible consequences, etc. 5. Finally, he is examined *viva voce* in all the different branches of medicine by all the Professors of the Faculty. The written answers to the questions are drawn up in the presence of an inspector, and no other help but a Latin dictionary is allowed. All the other examinations are public, and they have been hitherto in the Latin language, the explanations of the surgical operations excepted. The oral examination lasts a whole day for each candidate, every professor examining during the space of an hour or an hour and a half.

When the examination of the day is finished, the candidate and all the auditors leave the room, and the professors hold a council, in which they determine by vote the general character which is to be given to the candidate. There are three such general characters: *Laudabilis haud illaudabilis*, and *Non contemnendus*. The last named is seldom given, the candidate entitled to no other being commonly rejected; but it is proper to state, that the examination of the Medical Faculty is conducted in the most liberal spirit, and that greater notice is taken of the general knowledge and ability of the candidate than of refined subtleties and smaller deficiences. When the professors have agreed upon the general character, the candidate is called in, acquainted with the result, and an oath in the following terms is taken from him: "Gratulamur tibi de exhibito eruditionis specimine et speramus te eandem optimo modo in emolumentum societatis et proximi adhibiturum atque magis magisque aucturum nec non leges, quæ medicum et chirurgum obstringunt, tibi cognitas redditurum et impleturum, in cujus promissi sanctam observantiam te oportet dextrum porrigere." The testimony of having undergone the "examen medicum" is this: "anno [ ] die [ ] speciminibus scriptis et tentaminibus scriptis rite absolutis N. N. philæsophiæ et philologiæ in almo universitate Havniensi Candidatus et Medicino Studiosus examini medico chirurgico practico rigoroso se subjecit indeque (*Laudabilis* or *Haud illaudabilis*,) discessit."

The sum which the candidate has to pay for admission to the examination is very small, not amounting to more than twenty dollars (2*l*.,) of which the Dean receives five, the eldest professor five, and the youngest (as Secretary of the Faculty,) two dollars. Examinations takes place twice in the year, in the spring and in the autumn.

When the candidate has gone through the *examen*, he is immediately allowed to practice medicine and surgery wherever he pleases in the Danish states, the degree of doctor not being necessary to practice. The degree of M. D. is in Denmark but a literary honor, which only is required when any one aspires to a professorship at the university. It will from this at once be seen, how much the arrangements differ in this respect from those of the universities in Germany, and particularly in Prussia. If a foreigner wishes to practice as physician or surgeon in the Danish states, he must, besides being previously matriculated at another university, undergo a preliminary examination in the same branches of classical education that are required at the and "examen artium" and "examen philosophicum," and not until then is he admitted to the "examen medicum rigorosum."

### III. THE DEGREE OF DOCTOR OF MEDICINE.

Whoever wishes to take the degree of M. D. at the University of Copenhagen, must necessarily, 1. be matriculated at the university and have taken the "examen philosophicum;" 2. submitted to the "examen medicum" and have obtained the first character (laudabilis;) and 3. must be "Licentiate of Medicine." In order to become licentiate, he must write a medical dissertation in the Latin language, and deliver it to the Medical Faculty to be examined; when it is admitted to be well and scientifically written, the candidate must get it printed, together with the sentence passed upon it by the Faculty, and then defend it publicly, in the Latin language, in the hall of the University, against two professors of the Faculty appointed for this purpose, ("opponentes ordinarii,") and against any one of the auditory who may choose to question it. If the candidate defends his dissertation in an able manner, the Faculty announces it to the Royal Board of the University, which, with the King's permission, assigns the name of "Licentiatus Medicinæ" to him. Only those who are in public offices, or have acquired a celebrated name, are allowed to take the degree of doctor before being "Licentiati;" but, if that is the case, the degree of doctor is obtained by writing once more a Latin dissertation, and doing all the above-mentioned things over again, defending the essay publicly, etc.

It is to be remarked that much stress is laid upon the taking of the degree of licentiate and doctor, and that nobody is allowed to receive them without having written a really good essay, and given proofs of solid knowledge and a true scientific spirit in the composition and defence of it. The name of a Danish Doctor of Medicine is consequently held in much greater respect than that of a German; and many of the candidates have by their inaugural essays really contributed to the progress of science. At the public defence of the dissertations, which usually lasts a whole day, the candidate is allowed as assistant ("Respondens," as he is called,) who commonly is younger than the candidate, and whose name appears on the title page of the printed dissertation.

The sum which is paid for admittance to the degrees of licentiate and doctor is a small one; being for the first twenty-five dollars (2*l.* 10*s.*;) for the second, seventy dollars (7*l.*) of which the Dean at the Faculty receives forty dollars (4*l.*) The greatest expenses are incurred in the printing of the dissertations; about one hundred copies of which are distributed gratuitously to all the professors of the university, to the public libraries, etc.

The degree of doctor is held in such high esteem, that a special academical meeting is appointed for the occasion, and previously to the ceremony a program is issued, written by the Professor of the Latin language at the university, and accompanied by a note of the life of the candidate. At the inauguration, the Dean of the Medical Faculty ascends the chair, and, after a Latin speech, composed for the occasion, upon one or other fitting medical subject, he confers the degree, making use of the old symbols; the *book*, the *hat*, and the *kiss*. After this, the new-made doctor ascends the rostrum, and in a Latin speech returns thanks for the honor conferred on him.

Every one bearing the degree of doctor is entitled as well to aspire to a professorship as to give public or private lectures; and, if he makes application for a public appointment, he is, *cæteris paribus*, preferred to other candidates. He has also a certain rank in the state, for which however he must pay a certain annual tax. These are all the advantages of being a doctor of medicine in Denmark. The real value of the degree is only known to literary persons acquainted with the signification of the word and the thing; for it is the custom in Denmark, or elsewhere, to call every practitioner "doctor." As has been already stated, no one can get appointed to any of the public or royal medical offices in Denmark except those who have undergone the examination of the Medical Faculty at the university, although the degree of doctor is not required. But these offices are few in number, not amounting in all to more than twenty-three. They are as follows: five professors at the university; eleven county physicians; one physician of the Public Lunatic Asylum; two physicians of the court, (whose duty comprehends attendance on all the inferior officers and servants;) one physician of the poor in Aalberg in Jutland; one county physician in Iceland; one in the West Indies; one physician of the King. The prosecutor at the university, and the physician at the second hospital (the general) in Copenhagen, have both other surgical offices. The professor of midwifery and surgery at the university, J. S. Saxtorph, is also physician of the lying-in hospital; the professor of physiology, J. D. Herholdt, is the first surgeon of the navy; the professor of therapeutics, C. Bang, is physician of the first hospital (Fredrick's) in Copenhagen; the professor of anatomy, F. D. Eschricht, is physician of the Foundling-Hospital; and the Professor of Pharmacology and Forensic Medicine, the writer of this, is physician of the public Penitentiary. The first and the last offices are not always united with the professorships, but the others are always so. Of medical officers not royal there are three physicians of reserve and eight assistants (called candidates) at the hospital.

IV. SCIENTIFIC ESTABLISHMENT.

1. *The Dissecting Room and the Anatomico-Pathological Museum.*—The last mentioned is a very valuable collection of anatomical, pathological, and zootomical preparations, and contains about 1400 species. For the support of this establishment, 10,000 dollars (1000*l.*) are set apart, which are a portion of 294,000 dollars (29,000*l.*,) that were left as a legacy to public establishments by a rich Jewish merchant, Amsel Meyer. Another Anatomico-pathological Museum is at the College of Surgeons; and a third at the Veterinary School.

2. *The Royal Museum of Natural History.*—This was founded in the year 1796, by the union of several minor museums, and divided into two classes—zoology and mineralogy. The first consists of a collection of European birds, presented to the museum by Captain Woldicke; a collection of African birds, presented by Mr. Hancke, Danish consul at the Cape of Good Hope; of birds from South America, etc.; and a collection of insects from the most remote period, described by the celebrated Fabricius in his works on insects, fishes, reptiles, etc. The second collection (of minerals) is extremely valuable; the minerals are from Iceland, Feroe, Greenland, Hungary, Austria, England, etc. The museum is open to the public twice in the week.

3, *The Museum of Natural History at the University.*—This contains many zoophytes, insects, birds, etc., but more particularly minerals, which are used for the illustration of the lectures on mineralogy.

Besides these public museums of natural history, there are several private ones; as, for instance, one that belongs to the hereditary Prince Christian Frederik; one belonging to the apothecary Becker, etc.; but what deserves to be particularly mentioned here is a large collection of all sorts of animals belonging to a private "*Society for Promoting the Knowledge of Natural History*," which has been founded only three years, but consists of a very great number of members. The museum is open every Sunday to the members of the society and their families; and once in the year, during a fortnight, to the public. On every other Sunday in the winter a popular lecture is given alternately by Professor Eschricht at the university; and Schouw, professor of botany, upon some subject in natural history.

Another private society, which also has been founded lately, is that "*for the Promotion of the Natural Sciences*," which owes its origin to the celebrated Oersted, the greatest ornament of the university. This society, which has done much for the natural sciences in Denmark, has an excellent museum and maintains lecturers on the natural sciences in all the Danish provinces. Every other Sunday in winter popular lectures are given to a large audience of members by

Oersted or by Forchhammer, professor of mineralogy at the university, and a celebrated name in chemistry.

The writer of this Report possesses a good Phrenological Museum, consisting of natural skulls and casts, the last for the most part from Edinburgh.

4. *The Collection of Philosophical Instruments.*—This belongs to the university, and is very rich and valuable; it is under the superintendence of Oersted. The veterinary college has also a small collection of instruments.

5. *The Botanical Garden.*—This was founded in the year 1778, and differs in this respect from most other botanical gardens that it is situated in the middle of the town behind the palace of Charlottenborg, (the royal academy of arts.) Although it belongs to the university, it is principally supported by the king; and is undoubtedly one of the most extensive botanical gardens of Europe. The number of plants amounts to 9000, and from four to five thousand different seeds are annually sown. The garden is daily open to every student from eight to twelve, and from two to seven o'clock. To the garden belongs an excellent library, consisting of a rich store of botanical books, plants, herbaria, etc. The manuscripts, and a Herbarium of more than 20,000 species of the late celebrated Vahl, are the treasures of the establishment. The Professor of Botany at the university is inspector of the garden; the present is the celebrated T. W. Horneman, who has published a complete catalogue of the plants of the garden, accompanied with a botanical description, entitled, "Hortus regius Botanicus Hafniensis," in two volumes, and also a very valuable systematical description of all the plants of Denmark ("Plantelæn," 1821.) The celebrated botanical work with plates, entitled "Flora Danica," also continues to be published under his inspection.

6. *Public Libraries.*—Of these there are several; the following are the principal:

*a.* The *Royal Library.*—This was founded by King Frederick the Third, in 1665, and is supposed to be one of the first royal libraries in Europe, containing about 400,000 volumes. It consists of nine magnificent rooms. The largest of them is 260 feet in length, and contains every work of antiquity; around it is a beautiful gallery appropriated for the same use. An isolated part of it is the *Northern Library*, which has been arranged since the year 1781, and contains the whole literature of the Danish states, and very much of the Swedish. Few public libraries have a more extensive collection of manuscripts, atlasses, and engravings. The engravings are from the most ancient period, and are divided into three classes. The eldest collection consists of 47,228 engravings, bound in fifty-five large volumes, which were collected in the seventeenth century, under the reign of King Christian the Fifth, and are of the greatest antiquity. A later collection, formerly belonging to Mr. Wasserschleben, Privy Counsellor, to his Majesty, consists of 29,016 engravings, and is bound in 212 large volumes. According to a law passed in 1821, two copies of every book printed in the Danish states are to be delivered to the library. It is open every work-day from eleven to one o'clock, and in the reading room every one may, during these hours, read and make extracts from books; and they are also most liberally lent to any one who either is known, or procures a written security from some one who is so.

*b. The Library of the University.*—This is arranged in a spacious saloon over Trinity church, and contains about 100,000 volumes. The manuscripts, presented to this library by Arne Magnussen, are also very numerous, and illustrate the antiquity, geography, history and language of the northern nations. Independently of this extensive bequest of books, he left to the university a considerable legacy, the interest of which is appropriated to the extension of literature. The library is also much indebted to Rasts, the late celebrated linguist, and to Dr. Wallich, at Calcutta, for a fine collection of Oriental works, which are here deposited; it is indebted to the late Count Moltke, Minister of state, for a collection of works in natural history; and to the Counsellor of state, J. H. Schou, for a large collection of books belonging to the science of law. The voluminous manuscripts of Iceland are here deposited, and are very remarkable; but the greatest curiosity is one volume in Runic characters, which is the only book of this description in existence; it contains the most ancient laws of Denmark. The old seals and letters of Norway also deserve to be mentioned, and an ancient copy of the celebrated Edda. To this library also a copy of all Danish books must be delivered gratuitously. A reading-room is attached to the library, and is open daily, from ten to two o'clock; books are also lent from this library.

*c. The Classenian Library.*—This consists of 30,000 volumes, and possesses, in particular, books in mathematics, natural history, economy, geography, travels, etc. Major-general J. F. Classen, and his brother P. H. Classen, privy-counsellor to his Danish Majesty, were founders of this library. Amongst the valuable collection of books is a superb edition of botanical paintings in seventy volumes, by J. T. Herner, and which was presented by the present king, when prince-royal of Denmark. The library has a reading-room, and is open to the public four days in the week, from eleven to two o'clock, and books are lent, as in the other libraries.

*d. The Classenian Library for the use of Medical Men.*—This consists only of medical books, which already amount to four thousand, and are increasing considerably every year. It is supported by a bequest of the above-mentioned brothers, Classen, and by pecuniary contributions from the members of "*the Classenian Society of Literature for Medical Men;*" among whom the books and journals that are bought circulate, before they are deposited in the library. It is open to all medical men one day in the week from eleven to one, and books and journals are lent.

Besides these libraries, there is one at the Veterinary School, one at the Royal College of Surgeons, etc., besides many large private ones.

B. THE ROYAL COLLEGE OF SURGEONS, OR THE SURGICAL ACADEMY.

Surgery has since the year 1736, had its own school in Copenhagen. It formerly was called "Theatrum Anatomico-Chirurgicum;" but, in the year 1785, the institution was considerably enlarged, and got the above name, together with a new magnificent building, which was opened in 1788. This college, more particularly in the beginning, has contributed much to the progress of surgery in Denmark. Before its institution, surgeons and barbers were one and the same thing, and, of course, no well-bred young man or of the more respectable classes devoted himself to surgery.

All surgical offices, military and civil, are now only filled with gentlemen who have studied and passed their examination at this college.

The college consists of three ordinary professors, one adjunct, two lecturers (one in chemistry, another in anatomy,) four teachers, (who are called "surgeons of reserve," and are always young men, very often such as have been examined not more than two or three years before at the college,) and one candidate teacher.

The following are the names of the lecturers, and the departments taught by them respectively:

*Ordinary Professors.*

C. Fenger, (the surgeon of the king,) Surgery and Midwifery.
C. Withusen, (now general-director of the college,) Anatomy and Diseases of the Eyes.
A. Callisen, - - Surgical Pathology and Therapeutics.

*Adjunct Professor.*

J. C. J. H. Gundelach-Moller, Forensic Medicine and Clinical Surgery.

*The Lecturers.*

G. Scharling, - - - Chemistry.
C. L. F. Henck, - - - Anatomy.

*Surgeons of Reserve.*

M. Diorup, - - - - - Materia Medica.
J. F. C. E. Starck, - - Botany.
J. Rorby, - - - - Special branches of Surgery and Pathology.
J. Pape, - - - - - Special branches of Anatomy and Therapeutics.

*Candidate Teacher.*

J. E. Larsen, - - - Special branches of Surgery.

All the lectures are public; no private ones are allowed.

It will be seen that there is abundant means provided for teaching anatomy at the college, as well as at the Medical Faculty of the University. Indeed, Copenhagen is one of the places where this branch of medical science may be studied with the greatest advantage, not only on account of the able teachers here, but also because the student has the greatest facility and best opportunity of dissecting, both at the Faculty and at the college. In both, the dissecting rooms are open every work-day, and dead bodies are to be got at extremely small cost. From the hospitals the dead bodies of poor persons, who either die without any relations or with relations that do not care for them, are often to be had for no more than two English shillings. The standard price at the Faculty for a body is four shillings. Both institutions are, according to law, alternately and gratuitously furnished with the dead bodies of the criminals who die in the different public penitentiaries.

The same defect already noticed respecting the lectures at the Medical Faculty is also observable at the College. There are no lectures given on physiology, dietetics, morbid anatomy, on the diseases of the teeth, of the ears, of the mind, etc.; and very often the most important branches of surgical and medical knowledge, as, for instance, semeiotics, are left to the junior teachers, who of course want the necessary practical experience; faults which, as already mentioned, would be remedied, if the College of Surgeons was united with the Medical Faculty.

In order to show how small the remuneration is which in Denmark is given for scientific labors, I must here also notice the salaries of the lecturers at the College. The three professors get no more than 660 dollars (60*l.*) annually; the adjunct professor fifty dollars (5*l.*;) the lecturer in chemistry four hundred dollars (40*l.*;) the four surgeons of reserve, 144 dollars (14*l.*)

To the College belong, 1. a very good *Anatomical and Pathological Museum*, which has an inspector, and is open to the student twice in the week in the summer, from twelve to two o'clock, at which time the inspector, Mr. Ibsen, explains the preparations; 2. a *Library of Surgical and Medical Books*, of which Professor Callisen is librarian; and 3. a *Collection of Surgical Instruments*, which, however, is as yet very small.

To enable one to study and to be examined at the College of Surgeons, it is not necessary to be matriculated as "civis academicus" at the university, but the lectures are open to every one who pleases to attend them; and, when anybody wishes to be examined at the College, he petitions the professors, and delivers at the same time his *biography*, which indicates his native place, his age, parents, the school he has attended, his literary and medical education; and this must, of course, be accompanied by testimonials of having attended the lectures at the College, the Hospital, and the Clinics.

The candidate, when admitted, must either present to the College an anatomical preparation made by his own hand, or, instead of this, pay ten dollars, (1*l.*;) so that either nothing is paid in money or only this small sum. He must, in the first place, give written answers to a surgical, therapeutical, or medico-forensic question, in the Danish language; in the second place, he must make an anatomical dissection, (which commonly requires a whole day;) thirdly, he must examine a patient in the surgical clinic in Frederick's Hospital; and finally, he is examined, *viva voce*, in the Danish language, by the professors, the adjunct, and the lecturer in chemistry, during eight hours, which are distributed over three days. At the end of the examination on the third day, the candidate is called in, and is made acquainted with the particular character he has received; and, on being admitted, the following oath, in Danish, is taken by him: "After having publicly proved my medical and surgical knowledge, I do hereby promise and engage myself, with oath and hand, to the board of the College of Surgeons, that I shall always endeavor, in my function as practitioner, to employ my art zealously for the benefit of the community and my fellow-creatures; that I will take equally scrupulous care of the poor and the opulent, without any personal regard; strive to enlarge my knowledge, and learn and comply with the laws and ordinances that belong to my profession."

There are six general *characters* given at the examination, a number which certainly is too great, although in fact they are reduced to three principal ones, as every character (even the lowest,) entitles the candidate to practice medicine as well as surgery every where in the Danish states; but, of public offices, they can only get such as belong to the surgical department: yet these surgical offices surpass in number by far the medical ones. The royal public surgical offices amount to sixty-two military and eighty-four civil; but, of surgical offices that are not royal and very subaltern, there are ninety-four in the army and seven in the navy; besides an office for a surgeon of reserve at the second hospital in town, (the General Hospital,) for one at the Military High School, and offices for eight assistants ("surgical candidates") at the two hospitals. Nobody, who has only been examined at the College of Surgeons, is allowed to take the degree of doctor.

The number of students at the College was formerly from 130 to 150, but this number has also increased in later years, (now it amounts to 200, fifty of which number being without any academical education;) and, when it is considered that the surgical offices in Denmark are only filled by those who have been examined at the College; that these offices far surpass the medical ones in number; that the two examinations at the university (the "examen artium" and "examen philosophicum,") are not required at the College; that, furthermore, nothing, or (failing an anatomical preparation) only ten dollars (1*l*.,) are paid by the surgical candidates, it certainly is not to be wondered at that many more get examined at the College than at the Medical Faculty, and that even the greater part of those who submit themselves to the examination of the Faculty previously go through the examination at the College, in order to be entitled to make application for surgical offices. The folly of having two institutions where the same branches of the art are taught, the same examinations made, (differing only so far that the Latin language is used at the one, the Danish at the other,) is evident; and, were it only on this account, a reformation of the whole medical and surgical instruction in Denmark is loudly called for.

### C. THE MEDICAL PROFESSION IN DENMARK.

From what has been stated of the qualifications requisite for admission to the examinations at the Medical Faculty at the University, and at the College of Surgeons, and of the extent of knowledge required at both in all the different branches of medicine and surgery, it is evident that the state has done every thing in its power to secure the aid of able and enlightened men in an art upon which depend the health and life of its subjects. Every physician and surgeon in Denmark gets an education which qualifies him to maintain the dignity of his profession, as a worthy member of a class that is generally considered as one of the most respectable and most liberal. The Danish medical men are usually held in high esteem. We have seen how much the doctor of medicine deserves respect from his scientific acquirements; and Danish physicians and surgeons are so honored abroad, that very often Swedes come to Copenhagen in order Both treated by them. Mountebanks and quacks amongst the Danish medical men are likewise rare. This is the more to be wondered at, since the number of both physicians and surgeons is increasing greatly every year, in every part of the Danish dominions. In Copenhagen alone, with a population of 110,000, the number amounts already to 183, and that the condition, as well of those endowed with public offices as of mere private practitioners, is none of the most brilliant, will be evident from the following facts.

The salary which the state give to those in royal public medical or surgical offices is extremely small; to the surgical, 150, 200, 400 dollars, (15*l.*, 20*l.*, 40*l.*;) to the medical, 400, 600, 900 dollars, (40*l.*, 60*l.*, 90*l.*) according to the time of service. In order to gain a livelihood for his wife and children, the officer must devote all the hours of the day to practice, which in the country is burthensome in the highest degree; the county in which his office is, being often forty English miles in circumference, and every year younger colleagues are settling in his county, who take more and more of his practice from him. Still worse is the condition of the mere practitioner without any public office: he is not, as in England, paid for every visit to the patient, but gets his fee after his recovery; or, still more commonly, he attends families as "house-physician," paying visits every time a member of the family gets sick, and receiving his fee every new-year's day. This fee for the attendance of a whole year amounts usually to from twenty-five to thirty dollars (3*l.*;) and this even from families where there are many children, and where, upon the whole, perhaps only one month of the year has been without attendance. Only very few give fifty dollars (5*l.*;) and he is a *rara avis*, and falling only to the lot of the older and very celebrated physicians, who pays the exceedingly large fee of 100 dollars (12*l.*) on new-years day! For the care of a patient who is not attended by the medical men in quality of house-physician, he must think himself well paid when he gets ten or fifteen dollars (1*l.* or 1*l.* 10*s.*,) and this perhaps after an attendance of several months. Many, even of the higher classes, pay nothing at all, and on that account, like good politicians as they are, change their physician every year or every time they become sick.

There is certainly a legal charge fixed for every visit the medical man makes, and for every receipt he writes; but such is the custom of the country, that a physician dare never ask his due, for by so doing he would be accused of sordid avarice, and be obliged to yield his patient to one or other of his many brethren, and at last lose entirely what practice he had. Only in the country the medical men who are not paid are accustomed to give in their bills, but many of their patients bitterly complain of such a mode of proceeding; and yet the charge for traveling fifteen English miles, and in this manner employing at least eight hours, is no more than five dollars (half a crown!) The Danish physician is really, to say truth, most fortunate in this respect when one of his patients, particularly a wealthy one *dies!* for he is then always *requested* to give in his bill for his attendance; and he may then charge the heirs with a sufficient sum without risking objections, although this now and then happens, and he is, according to law, the first paid of all the creditors.

The miserable and unjust condition of the Danish medical men in all these respects need not be pointed out. It should not be left entirely to the arbitrement of patients to pay what they please: it should be a strict legal order for the medical man to deliver his bill according to a legal charge; it would not then, as now, be thought strange and self-interested to do so; the wealthier and more grateful patients might still, when so minded, give *more* than the legal demand; and, from so liberal a profession as the medical, the poor certainly would not have anything to fear by such an arrangement; for who would charge them? If, according to the present system, medical men, poor themselves, are sometimes obliged to charge or receive a fee from them, would this not happen much more seldom when they received a better and sure remuneration from the more opulent? As it is, the young medical man cannot gain a livelihood by practicing, and the elder ones must, by the *number* of patients, try to compensate for the smallness of the fees: and this is one of the reasons why the most celebrated and most able Danish practitioners, endowed with the most valuable experience, are unable to do anything towards the advancement of the science.

In order to lessen the probable want that may arise to the family of a medical man who dies in poverty, there exist two *benevolent medical societies* for the relief and support of the widows and children of deceased medical men; one in Copenhagen, another in the provinces.

An amiable trait in the character of the medical profession in Denmark is the harmony that exists between its members. It scarcely ever happens that one practitioner, from sordid motives, blames the conduct of the other at the sick bed: one never interferes with the practice of another; and, if one is called to a patient whom he supposes to be attended at the time by another, or who has his own house-physician, he never prescribes for the patient without previously having ascertained whether he is called according to the wish and the consent of his colleague, and without having consulted with him; if he is called contrary to the consent of his colleague, or without his knowledge, he refuses to prescribe at all. On the other hand, in dangerous or obstinate cases, it is very common for one physician to take another with him in consultation; and there is not one single instance on record where such consultations have not ended in the most friendly manner.

The harmony and the scientific intercourse amongst the medical men in the Danish capital are supported by two *medical Societies.*

1. The *Royal Medical Society.*—This was founded as early as the year 1772, and counts now amongst its members all the elder and younger literary and able physicians and surgeons in Denmark. It consists of honorary, native, ordinary, and foreign corresponding members. The present number of the native ordinary members is forty-five; amongst the foreign members (sixty-two) are the following Englishmen; M. G. Wilkinson, of Sunderland; Sir Astley Cooper, of London; Dr. John Thomson and Dr. Wishart, of Edinburgh; Mr. Wardrop, Mr. Howship, and Dr. Elliotson, of London. The society meets only in the winter season, and on every other Thursday, at seven o'clock. At the meetings, original essays, remarkable cases, etc., are read by one or more members, and medical matters, and particularly epidemic diseases, are discussed, etc. The most scientific and friendly spirit pervades these meetings, and although now more than sixty years have passed over the society, its zeal for the science continues unabated. It has special laws, which have lately been revised. The society has hitherto published seven volumes of its Transactions, ("Acta Societatis Regiæ Medicæ Hafnieusis,") but the greater part of later transactions have been published in the Danish medical journal, "Bibliothek for Læger," (the Physician's Library,) and (the custom of writing in Latin having ceased,) will in future continue to be so.

2. The *Philiatria.*—This is a medical society of younger date, having existed only four years: it consists only of the younger members of the profession in Copenhagen. It meets every Tuesday in the winter, at six o'clock; and its object is rather to discuss medical matters than to read essays, although this sometimes happens. It consists at present of thirty members.

From the existence and flourishing state of two such institutions in a town of only third rank, it may be inferred that a great love for science and literary pursuits exists in Denmark; and certainly the Danish medical man is thoroughly acquainted with the progress of medicine and the medical literature of all foreign countries. In this respect he does not yield to the medical men of any other country; and, from his necessary study of foreign languages, which are all known and spoken by well-educated Danes of the better classes, he even surpasses, in this particular, as well the English as the French and the Italians. In fact, medicine is the branch of science which has reached the highest degree of perfection in Denmark. The King's Library has a rich stock of medical works and takes in many foreign medical publications; the Medical Library, formerly noticed, is still richer in medicine, and subscribe to almost all the medical journals of Europe, the Italian alone excepted, which cannot be had without great difficulty. In the provinces also there are circulating medical libraries; and many medical men have good private libraries, and likewise subscribe to journals.

Notwithstanding this familiarity with medicine and its literature abroad, the medical men of Denmark are not prone to adopt new methods or new systems of medicine; and all the romantic and mystic hypotheses of Germany have been received with distrust, and have not been able to take root amongst us. We have never been either the first to adopt the new before it was sufficiently tried, nor the last to leave the old when its usefulness was proved beyond all question.

In their general methods of cure, the Danish medical men do not commonly use, but rather shun, heroic remedies; as true sons of Hippocrates, they follow his maxims in studying nature, and in endeavoring, in all their treatment, to obey it, and to sustain the *vires medicatrices*, not disturbing them by too active medicine. That, nevertheless, no extravagance in this respect is allowed to take place is proved by a golden book, by the late celebrated practitioner, Dr. Ludvig Bank, entitled "Praxis Medica," written in Latin, and once a standard of medical practice in Denmark. Nor is Denmark a home for quackery, being by far much less filled with quacks than England, France, or Germany. The rights of authorised medical men are strictly secured by laws which prohibit every species of quackery and unauthorised dispensation of medicine. In order to preserve the enforcement of these laws in all their vigor, all physicians and surgeons are bound to put their names and official function on every one of their written prescriptions; and an alphabetical list of all authorised medical men and their assistants is annually distributed among the apothecaries, chemists, and druggists, who are prohibited by a heavy fine from delivering medicine to any other but those who present a prescription signed by the name of a legalised medical man; and it is incumbent upon every assistant (amanuensis) of a practitioner to take his examination within two or three years from the time of his appointment as assistant. All the provincial medical officers and the state-physician in every town must, according to law, report every year to the highest medical authority in Denmark whether any quackery has made its appearance during the year at their place of residence.

The "highest medical authority" in Denmark is the Royal Board of Health. which resides in Copenhagen, and was instituted in the year 1803. According to the royal decree, it now always must consist of ten medical members, five physicians, five surgeons, and two apothecaries. Every thing relating to medicine, surgery, and pharmacy in Denmark, medical police, medical and surgical practice, the state of the apothecaries' shops, the behavior of midwives, etc., are under the superintendence of this Board of Health. Reports of epidemic diseases, of the state of health of the respective places, of remarkable cases, of unhappy accidents, of the quality of medicines that are sold by the apothecaries, of unauthorised practitioners, etc., the physicians, who again are bound to obtain like special reports from must annually be sent to this College of Health by the several county surgeons and the mere private practitioners in their counties. The College of Health decides also upon the doubtful mental state of criminals or the accusal, and has likewise the privilege of making proposals to the king about the filling of vacant medical and surgical offices, on which account all applications for such vacant offices are first sent to it; and so great is the impartiality of the king, that he scarcely ever omits to ask the opinion of the College of Health, or acts contrary to it by listening to patrons or friends of the candidates for an office. It is but justice alone to state that the College acts with the greatest impartiality, weighs the respective merits of the many candidates in doubtful cases, leaving the final decision to the king. The only objection that might be made in this respect is that too much stress is laid upon the *age* of the candidates for such offices. The College of Health is also bound, in conjunction with the *Town* physician, to visit the shops of the apothecaries once a year (in the autumn,) in order to examine the quality and goodness of all the medicine on sale; on which occasion also many chemical analysis are made.

The principal administration of the functions of the College of Health is undertaken by the Dean, who is annually changed, and by turns chosen among the medical and surgical members of the College. The College assembles once a fortnight (ever other Saturday at six o'clock,) in order to examine and decide on the cases that have been laid before it; and these, in the interim, circulate among the members, all of whom, with the dean at the head, are bound to vote upon them.

It is remarkable that the members of this College, although really working hard in the fulfilling of their duties, and although having annually about six hundred different cases to decide upon, do not enjoy any pecuniary emolument or salary for their labors; the dean alone receiving, the year he is in office, 400 dollars (40*l*.,) and his secretary 200 dollars (20*l*.) It is also a defect in the organization of the College of Health that it cannot refer directly to the king, but that all its decisions and proposals go through the royal Chancery; an arrangement that necessarily must damp the activity and the influence of the College; the Chancery, although, not possessing one single man acquainted with medical affairs, often deciding contrary to the opinion of the College.—*Ib.* C. O.

*Copenhagen, March* 17, 1836.

14. *Case of Poisoning by Arsenic, effectually treated with the Hydrated Peroxide of Iron.* By J. R. Chilton, M. D., New York.—The patient was a young lady, who had been in the habit of taking calcined magnesia for heartburn. A paper containing powdered white arsenic, labelled, had, without her knowledge, been placed for safety in the same drawer in which she kept the magnesia. She opened the paper containing the arsenic, without seeing the label, and put about a tea-spoonful of it in her mouth in the dry state, (the way she usually took the magnesia) and did not perceive her mistake till after she had swallowed, as near as could be ascertained, a fourth part of it.

About 15 minutes had elapsed from the time she took it, till I saw her. An emetic of ipecac had been administered, which only caused her to throw up a little watery matter.

I found her with her strength much prostrated and complaining of a burning sensation at the stomach, and a feeling in her throat as if she would be suffocated.

It rather singularly happened that at the time I was called to see the case, I was engaged in preparing a quantity of the oxide of iron, to have it in readiness to test the beneficial effects which have of late been attributed to it, in such cases. I took with me about an ounce of it, while yet moist on the filter, and mingled it with water, and gave her half of it; finding that the stomach retained it. I gave the other half, and commenced kneading the abdomen over the region of the stomach, under the idea that as the stomach was in a torpid state, and the oxide of iron being insoluble in water, and of great weight, it would soon settle to the lower parts, and therefore there might be particles of the arsenic lodged in the coat of the stomach which would not otherwise come in contact with the preparation. This kneading, although it caused considerable pain, was persisted in for some minutes, after which she was allowed to remain quiet. No unpleasant symptoms followed, until about four hours after, when she complained of griping pains in the bowels. I administered a dose of magnesia, which operated freely; and the next day found her free from pain, and only laboring under a little debility, which in the course of a day or two she entirely recovered from.—*Med. and Surg. Jour.*

15. *On the Medicinal Properties of Creosote.* By J. Elliottson, M. D., etc.—In the early part of 1834, M. Reichenbach discovered a new principle in pyroligneous acid and all the tars, which he called

Creosote, from its property of preserving animal matter; he regarded it as a cure for various diseases. Dr. E. commenced a trial with it at St. Thomas's Hospital, in July, 1834, in cases of phthisis and epilepsy. The medicine proved stimulating. If the first dose exceeded two or three drops, nausea, vomiting, vertigo, headache, and heat of the head, generally followed; but if the dose was at first only one or two drops, many patients bore a gradual increase of it to six, ten, or twenty, without unpleasant effect. Some patients will not bear more than a fraction of a drop of creosote, while Dr. E. knew one lady who steadily augmented her dose to forty drops before it disagreed with her; but the addition of a single drop beyond this produced extreme giddiness, insensibility, and vomiting, followed by headache for several days. It appears more likely to disagree if not well diluted, though the longer it is given, the less dilution frequently is necessary. At first every drop usually requires about half an ounce of water, and few persons can take many drops in much less than half a pint, without experiencing at least considerable heat in the tongue especially, and in the pharynx, œsophagus, and stomach. Dr. E's. trials with it internally in phthisis "were perfectly unsuccessful." It is no remedy for tubercles; but, where only a single ulcer exists, or but a small number are in the lungs, and there is no disposition to further tubercular formation, it is very useful.

"One young gentleman, with a large solitary cavity in his left lung, has completely recovered, and not the slighest morbid condition is discoverable by the ear. In bronchorrhœa, or that state of the bronchial mucus membrane which consists in a profuse secretion without inflammation, I have seen its inhalation of essential service. In one instance of this affection, in which the expectoration was extremely offensive, the cure was very rapid. In asthma also, dependent upon morbid excitability of the bronchial membrane, its inhalation is often useful. Even where it agrees perfectly well, the inhalation frequently induces a heat of the tip of the tongue." (p. 221.)

A few epileptical patients for a time had mild, or fits, and at longer intervals, from this remedy; but, except in one or two instances, the disease returned with its former severity, and sometimes appeared to be aggravated. The tranquilizing effect of creosote in some instances of epilepsy encouraged Dr. E. to try it in neuralgia, hysteria, and extreme nervousness; and in this class of cases it frequently succeeded. In rheumatic neuralgia, not inflammatory, D. E. imagines the remedy is most successful. The morbid excitability of nervous persons has been frequently abated by it in a remarkable manner; but in this description of persons, we are advised to begin with no more than a half a drop, as occasionally more at first produces excitement of the head. Palpitation depending upon mere morbid excitability of the heart has yielded to it far more than other remedies. The "extraordinary power" which the creosote possesses, of arresting vomiting, when not dependent on inflammation or structural disease, Dr. E. "considers to be perfectly established." During inflammation, the stimulant power of the medicine does harm. If structural disease exists, it aggravates pain, even if it arrest the vomiting. A few persons are so disgusted with the smell of tar, that any attempt to take the creosote makes them sick; but, with these exceptions, Dr.

E. "knows no other medicine at all to be compared with it in arresting vomiting." He has often seen it succeed after the failure of prussic acid, the most powerful remedy he was previously acquainted with in such cases. In different cases of vomiting different doses are required. In colic and enteritis, it arrests the vomiting long before the bowels are opened, and purgatives are thus retained which were before rejected. Dr. E. has tried it only in one case of vomiting from pregnancy, and with success. In sea sickness also it has been found advantageous, but at present upon a small scale. Its power, we are assured, is equally great over nausea. When properly given in cases of nausea or vomiting, without inflammation or structural disease of the stomach Dr. E. has only known it to fail in one remarkable case, which he relates. Having ascertained this power, experiments were made to determine whether, like prussic acid, creosote would prevent other medicines from exciting nausea and vomiting. The result is thus confidently expressed:

"I find it, by daily experience, even to surpass prussic acid in this particular. I have enabled the stomach to bear hydriodate of potass, sulphate of copper, sulphate of iron, and many diuretics, etc., in much larger quantities than those previously rejected. Just as I have often seen it arrest vomiting where prussic acid had failed, so I have seen it enable the stomach to bear medicines when they had been rejected in spite of prussic acid." (p. 230.)

No action is produced upon the bowels by the creosote. It sometimes augments the quantity of urine. Dr. E. once saw it, in doses of a minim three times a day, cause micturition nine times in an hour. In another case, in doses of three minims, it produced severe strangury. In three cases of diabetes, it was thought to do good. Dr. E. speaks favorably, too, of its external application in ulcers that require a stimulus, and in sloughs with offensive discharge. In pruritus prodicis, in toothache applied pure, and in porrigo, it has sometimes been found serviceable. Dr. E. adds, in a proscript, that he has witnessed "its extraordinary power over nausea and vomiting, in at least fifty more cases, in which the stomach was neither inflamed or diseased." In two cases of glanders contracted from a glandered horse, "the sedulous injection of a weak solution of creosote up the nostril, removed the whole of the symptoms in a few weeks."

So far we have confined ourselves to an abstract of Dr. E's. warm eulogiums respecting the creosote, but we cannot quit the subject without observing that, in other and equally judicious hands, with all the required conditions present in the case in which it was given, it has fallen very far short of the virtues ascribed to it in his paper. We sincerely hope that further experience may confirm Dr. E's. opinions: we fear, nay, we believe, it will not; and that, even in those cases—nausea and vomiting, with inflammation or organic disease,—in which he much extols its powers, the result of accumulated evidence will be, that it sometimes succeeds, more frequently fails, and is even occasionally injurious. The creosote is at least not to be trifled with, with impunity, and we caution practitioners in general, from what we know of its effects, to prescribe it with great circumspection.—*Brit. and For. Med. Review, July* 1836.—*Ib.*

